# Dependence of the Cyanobacterium *Prochlorococcus* on Hydrogen Peroxide Scavenging Microbes for Growth at the Ocean's Surface

**DOI:** 10.1371/journal.pone.0016805

**Published:** 2011-02-03

**Authors:** J. Jeffrey Morris, Zackary I. Johnson, Martin J. Szul, Martin Keller, Erik R. Zinser

**Affiliations:** 1 Department of Microbiology, University of Tennessee, Knoxville, Tennessee, United States of America; 2 Nicholas School of the Environment, Duke University Marine Laboratory, Beaufort, North Carolina, United States of America; 3 Oak Ridge National Laboratory, Oak Ridge, Tennessee, United States of America; Universidad Miguel Hernandez, Spain

## Abstract

The phytoplankton community in the oligotrophic open ocean is numerically dominated by the cyanobacterium *Prochlorococcus*, accounting for approximately half of all photosynthesis. In the illuminated euphotic zone where *Prochlorococcus* grows, reactive oxygen species are continuously generated via photochemical reactions with dissolved organic matter. However, *Prochlorococcus* genomes lack catalase and additional protective mechanisms common in other aerobes, and this genus is highly susceptible to oxidative damage from hydrogen peroxide (HOOH). In this study we showed that the extant microbial community plays a vital, previously unrecognized role in cross-protecting *Prochlorococcus* from oxidative damage in the surface mixed layer of the oligotrophic ocean. Microbes are the primary HOOH sink in marine systems, and in the absence of the microbial community, surface waters in the Atlantic and Pacific Ocean accumulated HOOH to concentrations that were lethal for *Prochlorococcus* cultures. In laboratory experiments with the marine heterotroph *Alteromonas* sp., serving as a proxy for the natural community of HOOH-degrading microbes, bacterial depletion of HOOH from the extracellular milieu prevented oxidative damage to the cell envelope and photosystems of co-cultured *Prochlorococcus*, and facilitated the growth of *Prochlorococcus* at ecologically-relevant cell concentrations. Curiously, the more recently evolved lineages of *Prochlorococcus* that exploit the surface mixed layer niche were also the most sensitive to HOOH. The genomic streamlining of these evolved lineages during adaptation to the high-light exposed upper euphotic zone thus appears to be coincident with an acquired dependency on the extant HOOH-consuming community. These results underscore the importance of (indirect) biotic interactions in establishing niche boundaries, and highlight the impacts that community-level responses to stress may have in the ecological and evolutionary outcomes for co-existing species.

## Introduction

The open ocean is the largest biome on the surface of the earth, but due to its distance from coastal and deep ocean sediments, is also one of the most oligotrophic. Nutrient scarcity is especially prevalent in the surface mixed layers of highly stratified systems, with inputs of nutrients often restricted to new production (e.g. nitrogen fixation) or atmospheric (dust) deposition [1]. Microbes dominate biomass in this “wet desert” [2] and the most abundant phytoplankter in the tropics and subtropics (∼40°N to 40°S latitude) is the unicellular cyanobacterium, *Prochlorococcus* [3]. This oligotrophic specialist has the smallest cell size (0.4 to 1.2 µm in diameter) and genome (1.7–2.5×10^6^ bp) [4], [5] of any known photoautotroph. The small cell size results in a superior surface to volume ratio that is believed to provide a key advantage in nutrient scavenging versus larger competitors [6]. The small genome size, together with a reliance on sulfo- rather than phospholipids [7], greatly diminishes its cellular P quota, providing an additional advantage over larger competitors. As a result of these and other adaptations, *Prochlorococcus* populations span the entire euphotic zone, often exceeding 10^5^ cells mL^−1^, and due to this numerical dominance have been credited for roughly half of all photosynthesis in the oceans [8]–[12].

Genetically distinct ecotypes of *Prochlorococcus* partition the oligotrophic euphotic zone niche with respect to depth and latitude, in response to gradients of light and temperature, respectively. The upper euphotic zone is dominated by two closely-related ecotypes, eMED4 (“e” for ecotype, “MED4” for the type strain of the lineage) and eMIT9312, which are high-light adapted (HL), while the lower euphotic zone is dominated by low-light adapted (LL) ecotypes that include eNATL2A, eMIT9313, and eSS120 [13]–[17]. In seasonally-stratified regions of the subtropics, deep mixing events facilitate an invasion of the surface mixed layer by the LL ecotype eNATL2A [17], [18], which in certain instances may lead to numerical dominance of the LL ecotypes in the mixed layer [19]. The high irradiances found near the ocean surface restrict the growth of eNATL2A [17] as well as the other LL ecotypes [16] but eNATL2A appears unique amongst the LL ecotypes in its ability to survive temporary exposures to high light, such as would be experienced during deep vertical mixing events [18]. The two HL ecotypes further partition the upper euphotic zone by latitude: the eMIT9312 ecotype dominates the middle band from 30° N to 30° S latitude, while eMED4 dominates the higher latitudes at the extremes of *Prochlorococcus'* distribution [20]. This latitudinal niche partitioning is driven primarily by ocean temperature [17]–[21], and is consistent with the growth properties of cultured ecotype representatives [20].

The pattern of diversification within the *Prochlorococcus* lineage is consistent with an evolutionary progression from LL ancestors restricted to the deep euphotic zone, towards HL strains able to exploit the high light niche in the surface mixed layer. The eMIT9313 ecotype is the earliest branching lineage from the last common ancestor of *Prochlorococcus* and *Synechococcus*, and like all *Prochlorococcus* contains pigments that optimize the cells for the utilization of the blue light wavelengths that penetrate deepest into the euphotic zone [22]. The LL lineages are polyphyletic, with the eNATL2A ecotype the most recently derived. Based on reconstructions of *Prochlorococcus* evolution from molecular phylogenies along with ecological observations, the emergence of the eNATL2A lineage coincided with the ability of *Prochlorococcus* to invade the surface mixed layer, albeit only in instances of deep vertical mixing that minimize the lengths of exposure to high light [17]. Acquisition of a DNA photolyase and an elevated number of high light inducible proteins may have been responsible for this adaptation for high light tolerance [23]. With the emergence of the eMED4 and eMIT9312 “true” high light ecotypes – the most derived lineages – *Prochlorococcus* gained a sustained presence in the mixed layer, irrespective of mixed layer depth. The HL ecotypes have a common “core” of ∼100 genes not found in the LL ecotypes, and many of these may be responsible for the exploitation of high light near the surface [23].

In addition to the high light and low nutrient stresses associated with the oligotrophic surface mixed layer, the invasion of the mixed layer by *Prochlorococcus* may have also involved elevated oxidative stress. While hydrogen peroxide (HOOH) is ubiquitous in the ocean, the photooxidation of dissolved organic carbon (DOC) by sunlight [24], particularly by light in the UV range [25], results in near-surface HOOH maxima. Along with iron, HOOH is enriched in rainwater [26]–[28], and the combination of the two may cause periodic oxidative stress in marine organisms via the generation of highly reactive hydroxyl radicals (OH^•^) by the Fenton reaction [29], [30]. HOOH has been shown to inhibit the growth of diverse marine algae, including cyanobacteria, macro- and microscopic chlorophytes, diatoms, and coral-associated zooxanthellae [31]–[36], albeit at levels higher than those typically measured in pelagic waters.

In prior work, we demonstrated that *Prochlorococcus* was dependent on “helper” heterotrophic bacteria to thrive in dilute laboratory cultures [37]. Many heterotroph strains were capable of helping *Prochlorococcus*, including members of the α- and γ-Proteobacteria and the Bacteriodetes cluster, suggesting the mechanism(s) are common bacterial activities. In these experiments, the helping phenomenon occurred when *Prochlorococcus* and heterotrophic bacteria were inoculated at ecologically relevant concentrations, suggesting that the helping mechanism might also play an important role in natural communities. Preliminary evidence suggested the helper phenotype was associated with protection from HOOH, compensating for *Prochlorococcus*' conspicuously diminished suite of antioxidant genes (e.g. catalase) compared to other aerobes [4], [38]. In this study, we were driven by the following questions regarding the heterotroph/*Prochlorococcus* interactions: is the removal of HOOH from the medium necessary and sufficient for the helping phenotype? Does this mechanism of protection have relevance to natural communities in the open ocean? How universal is the dependency on helpers for the growth of the *Prochlorococcus* lineages? Finally, what does the apparent loss of endogenous HOOH-protection mechanisms imply regarding the genomic streamlining of the *Prochlorococcus* genus?

## Results

### HOOH removal is necessary and sufficient for the helping phenotype

From our prior work, we hypothesized that heterotrophs help *Prochlorococcus* by the removal of HOOH from the medium [37]. To test this hypothesis we began by analyzing the impacts of *Prochlorococcus* and heterotrophic growth on the medium chemistry. Standard Pro99 medium prepared with filtered, autoclaved seawater was found to contain approximately 0.2±0.02 µM HOOH. Axenic cultures of *Prochlorococcus* UH18301 (a novel isolate of the eMIT9312 ecotype, Table S1) depleted medium HOOH either very slowly or not at all, depending on the initial inoculum (Figure S1). Compared to these axenic controls, however, co-cultures with the “helper” strain *Alteromonas* sp. EZ55 rapidly depleted this HOOH to below detection (<0.01 µM) (Figure S1). In contrast, pH, dissolved oxygen, bicarbonate, and dissolved organic carbon were not significantly different in axenic versus mixed cultures of *Prochlorococcus* during the first week of culture (t-tests for individual data points, df = 4, p>0.05, Figure S2). While CO_2_ concentration was different in mixed versus axenic *Prochlorococcus* cultures, this potential carbon source for *Prochlorococcus* was *less* abundant in co-cultures as a consequence of slightly elevated pH (Figure S2, C and E). While EZ55 on its own had the predicted effect of lowering O_2_ concentrations, and raising inorganic carbon concentrations, this change was relatively minor, short-lived, and not reflected in a difference between axenic UH18301 and co-cultures with EZ55 (Figure S2). Thus, of all the potential means by which helpers may benefit *Prochlorococcus* through their modification of the bulk medium, whether by adding a nutrient or removing a growth inhibitor, the removal of HOOH was the only one consistent with a beneficial effect for *Prochlorococcus.*

All confirmed helpers are catalase-positive [37], but the helping phenotype could not be unambiguously assigned to the catalase activity of these bacteria. While insertional inactivation of catalase in *Alteromonas* sp. EZ55, *Vibrio fischeri* ES114, and *Silicibacter lacuscaerulensis* ITI-1157 resulted in the loss of ability to help *Prochlorococcus* growth on agar media ([37], and data not shown), the results in liquid media were less clear. Catalase mutants of EZ55 and ES114 were still capable of helping dilute liquid cultures of *Prochlorococcus*, but they also showed negligible loss of HOOH scavenging ability in liquid (Table S2). Redundant HOOH scavenging pathways are common in aerobic heterotrophs [39], [40], and these are likely to maintain the heterotrophs in a help-competent state, at least for experiments in liquid (semisolid agar media may have higher HOOH levels that do require active catalase). In contrast, the *S. lacuscaerulensis* catalase mutant was unable to help *Prochlorococcus* in liquid, but by an unknown mechanism actually raised HOOH levels in culture media relative to sterile controls (Table S2). Studies with purified bovine liver catalase likewise provided ambiguous results. While catalase provided some benefit to UH18301 in comparison to untreated, axenic controls, it was clearly inferior in comparison to co-culture with bacterial helpers (Table S2). Catalase is photo-inactivated ([41] and Figure S3), and heat-killed catalase resulted in higher *Prochlorococcus* mortality than unamended controls (Table S2). Thus, light-driven degradation of catalase to toxic end products may account for these ambiguous results.

**Figure 1:**
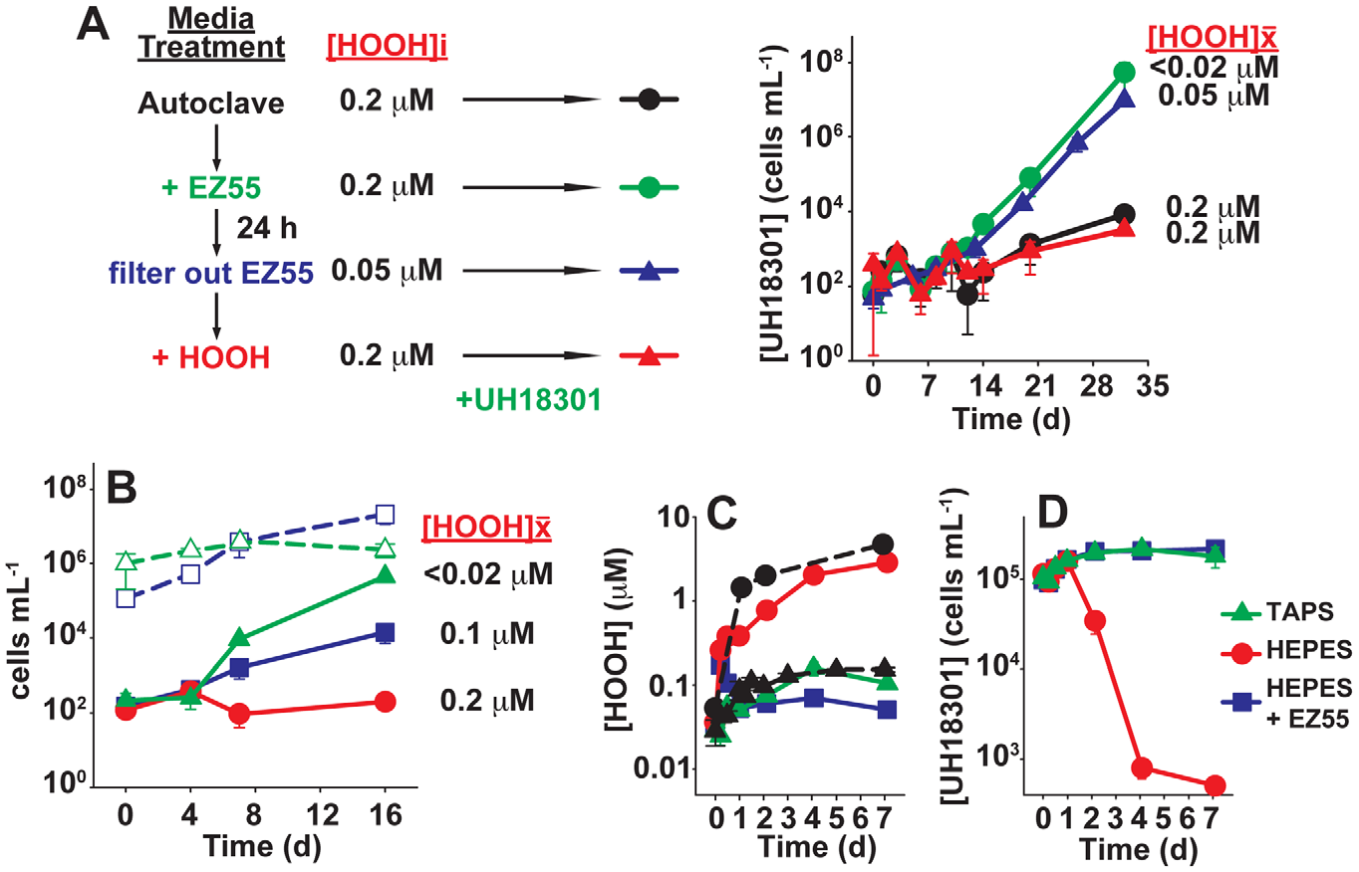
Removal of HOOH from media facilitates *Prochlorococcus* growth. A) Effects of pre-treatment of Pro99 medium on growth of dilute (initially ≈100 cells mL^−1^) axenic *Prochlorococcus* UH18301 cultures grown under low light. Prior to inoculation of UH18301, medium was autoclaved (black circles), pre-inoculated with 10^6^ cells ml^−1^ EZ55 (green circles), pre-incubated for 24 hours followed by removal of EZ55 via filtration (blue triangles), and then supplemented with HOOH to restore its initial concentration (red triangles). [HOOH]i, HOOH concentration at the time of inoculation; [HOOH]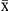, mean daily HOOH exposure, obtained by integrating [HOOH] over the first 7 d and dividing by the elapsed time. B) Growth kinetics of dilute *Prochlorococcus* VOL1 under high light in axenic cultures (red circles) and in co-culture with more concentrated *Prochlorococcus* VOL7 (solid blue squares) or EZ55 (solid green triangles). Also shown are the kinetics of the co-cultured VOL7 (open blue squares, dashed line) and EZ55 (open green triangles, dashed line). Error bars are the standard errors of 3 biological replicates. C) HOOH accumulation in high light-exposed UH18301-inoculated seawater containing 3.75 mM HEPES either with (blue squares) or without (red circles) EZ55, compared to an axenic control grown in media with 3.75 mM of the non-HOOH producing buffer TAPS (green triangles). Dashed lines, sterile media containing HEPES (black circles) or TAPS (black triangles). D) Cell concentrations over time in buffer-treated cultures. Symbols represent the same cultures as in 1C.

Because complications obscured the results of the catalase studies, we confirmed the role of HOOH scavenging in the helping phenotype by another line of experimentation, which was to manipulate the HOOH and heterotroph activities in media prior to inoculation with *Prochlorococcus*. Dilute (∼100 cells mL^−1^) axenic *Prochlorococcus* UH18301 was unable to lower the initial HOOH of the autoclaved medium, resulting in a mean daily exposure (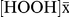) of 0.2 µM, and consequently exhibited very poor growth over this period (Figure 1A). In contrast, pre-inoculation of the medium with the EZ55 helper strain resulted in robust growth of UH18301. This significant growth improvement was associated with a much lower 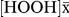 (<0.02 µM) as a result of the activity of EZ55. The activity of EZ55 was sufficient to pre-condition the medium for *Prochlorococcus* growth, as evidenced by the robust UH18301 growth in media pre-incubated with EZ55 for 24 hours, followed by removal of the EZ55 cells via filtration prior to inoculation of the *Prochlorococcus* cells. As for the co-culture treatment, this pre-conditioning treatment also resulted in a significantly lower [HOOH]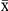 for *Prochlorococcus*. Filtration of the EZ55 did not appear to release intracellular components into the medium, as no detectable hydroperoxidase or alkaline phosphatase activity remained in the filtrates, in contrast to the unfiltered cultures themselves (Figure S4). Finally, if HOOH was added back to the EZ55-conditioned medium, restoring the HOOH concentration to 0.2±0.02 µM, the enhanced growth effect of the EZ55 preconditioning step on *Prochlorococcus* growth was lost (Figure 1A). This indicates that any activity of EZ55 other than the removal of HOOH is not sufficient to explain the helping phenotype of these cells.

The ability of axenic *Prochlorococcus* to grow in concentrated but not dilute cultures [37] suggests that *Prochlorococcus* can help itself in a density-dependent manner. To confirm this, we conducted co-culture experiments between two strains of *Prochlorococcus* distinguishable by quantitative PCR. Axenic VOL1 (a streptomycin-resistant [SmR] mutant of MIT9215, Table S1) had a minimal influence on the medium [HOOH] and was incapable of growth from 100 cells mL^−1^ (Figure 1B and [37]). However, when co-cultured with 10^5^ cells mL^−1^ of VOL7 (a SmR mutant of MED4, Table S1), VOL1 survived and grew at approximately the same rate as VOL7 (Figure 1B). VOL1 grew slower in co-culture with VOL7 compared to the heterotroph, EZ55, and consistently, the ability of VOL7 to remove HOOH from the medium was also substantially lower than EZ55 (Figure 1B). These observations support the hypothesis that *Prochlorococcus* is a poor scavenger of exogenous HOOH, but by mass-action at high densities can draw down HOOH to sub-lethal levels.

The experiments described above involved exposing dilute *Prochlorococcus* cultures to the elevated HOOH of autoclaved media. However, the poor HOOH scavenging capacity of *Prochlorococcus* and its dependency on help is also apparent when ecologically-relevant concentrations of cells (∼10^5^ cells mL^−1^) are challenged with HOOH that accumulates gradually in the medium. To avoid potential influences of the concentrated Fe, N and P in growth media, we performed these experiments in sterile unamended seawater. In the presence of light, HEPES buffer generates HOOH [42], [43], and under high light (∼250 µmol quanta m^−2^ s^−1^), the rate of HOOH generation in sterile seawater containing 3.75 mM HEPES was approximately 0.4 µM d^−1^ (Figure 1C). Axenic *Prochlorococcus* UH18301 was unable to prevent HOOH accumulation to lethal levels (Figure 1C), resulting in a rapid decline in cell counts (Figure 1D). The same outcome was observed even when the HEPES concentration was lowered to reduce the rate of HOOH production by 75% (Figure S5). However, if the *Prochlorococcus* cultures also contained EZ55, or if the HEPES buffer was substituted with the buffer TAPS, which generates much less HOOH (Figure 1C), HOOH concentrations were maintained at or below 0.1 µM, and subsequently *Prochlorococcus* cells survived, doubling about once before running out of nutrients in this unamended seawater medium (Figure 1D).

### Ecologically relevant levels of HOOH inhibit axenic *Prochlorococcus*

**Figure 2:**
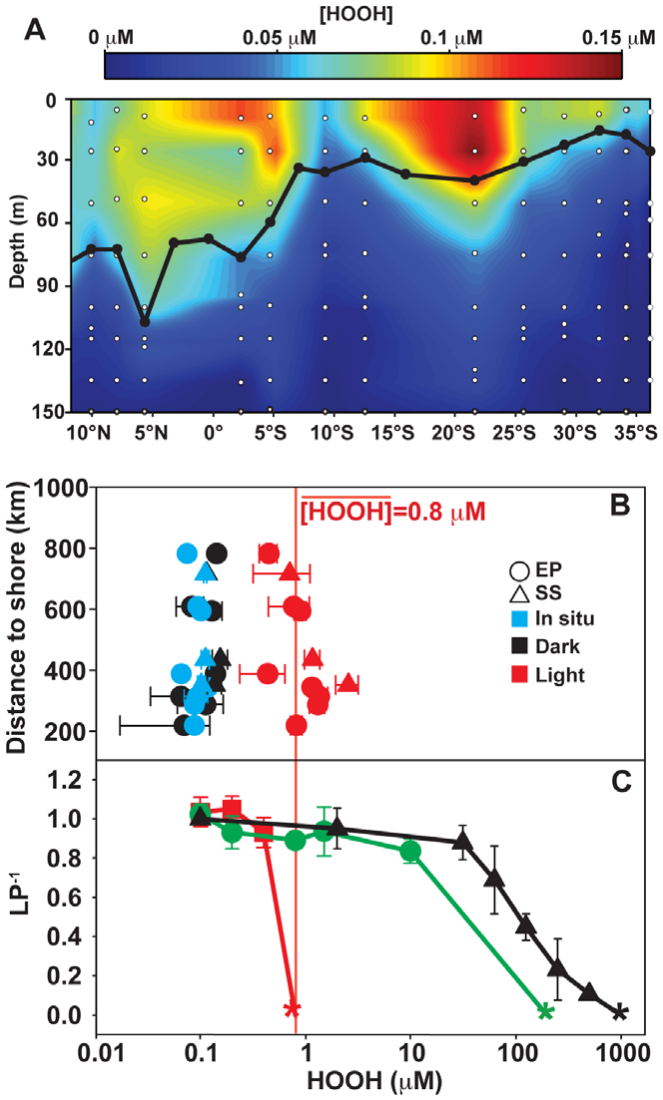
Removal of HOOH is necessary for *Prochlorococcus* survival at the ocean's surface. A) [HOOH] in the euphotic zone along a transect from Hawai'i to Australia, Jan-Feb 2007. Sampled depths (open circles) used in the linear interpolation of HOOH, and surface mixed layer depth (black line) are reported. B) Effect of solar irradiation on [HOOH] of seawater. “Light” and “Dark” indicate HOOH concentrations after 24 h incubations as described in Methods. Vertical line represents the mean [HOOH] in light incubations, represented by the SMC value as described in the text. EP, Eastern Pacific stations; SS, Sargasso Sea stations. C) Effect of HOOH exposure on the long-term growth of *Prochlorococcus* UH18301 cultures. Lag phase duration, expressed as the inverse ratio of each sample to a no added HOOH control (LP^−1^) as a function of HOOH dosage with (green circles) or without (red squares) EZ55. Black triangles, LP^−1^ of EZ55 in YT3 medium; *, all replicate cultures failed to show detectable growth after 60 d. Error bars are the standard error of three biological replicates. Cultures were grown under low light with an initial inoculum of ∼10^5^ cells mL^−1^.

For the experiments described above, initial HOOH concentrations were between ∼0–0.2 µM. Importantly, this is well within the range observed *in situ* in the ocean euphotic zone [26], [44]–[49], and as these experiments also involved ecologically-relevant concentrations of *Prochlorococcus* and helper, our results may reflect important interactions that occur in natural communities. The primary sources of HOOH in the ocean are rainfall events [26]–[28], [32] and photochemical (especially UV) degradation of dissolved organic carbon [25], [50], [51]. Accordingly, the highest concentrations of HOOH in the oceans are in the upper euphotic zone, which we confirmed in a 2007 meridional transect in the Pacific Ocean (Figure 2A and Table S3). With a few exceptions, the concentration of HOOH in the surface mixed layer ranged from 0.075–0.15 µM, whereas the concentration rapidly dropped below the mixed layer and fell below the limit of detection (<0.01 µM). From these measurements we conclude that the greatest threat of HOOH-mediated oxidative damage for *Prochlorococcus* is within the mixed layer.

HOOH concentrations in the ocean are instantaneous measurements that reflect competing rates of production and removal. While sunlight is the primary source of HOOH, the primary sink is the microflora [52], [53]. To investigate the role that the extant microbial community plays in maintaining HOOH concentrations at the surface within the tolerable range for *Prochlorococcus*, we examined the effect that removal of this primary sink had on the local concentrations of HOOH. In total, 11 Pacific and Atlantic open ocean stations were chosen for analysis (Table S3), each with mixed layer HOOH concentrations of ∼0.1 µM (Figure 2B) and *Prochlorococcus* present in typical abundance (10^4^–10^5^ cells mL^−1^, data not shown). Extant microbiota were removed from surface seawater via 0.2 µM filtration, and samples were assessed for gross HOOH abiotic production in the absence of the sink. After 24 hours incubation, dark controls were not significantly different than the in situ values (paired t-test, p>0.05), whereas the experimental samples incubated at approximately 80% of surface intensity sunlight (visible + UV), simulating exposure at 5 m depth, showed dramatic increases in HOOH (Figure 2B). This maximal concentration varied from station to station but had a mean of 0.8 µM±0.6 µM. In the experiments that follow we refer to this HOOH concentration as the surface monoculture (SMC) challenge, as it represents the hypothetical situation where *Prochlorococcus* is exposed to naturally generated HOOH in the surface mixed layer in the absence of HOOH-removing helpers.

In the absence of helpers, the SMC concentration of HOOH was lethal to all three of the mixed layer ecotypes. While concentrations of HOOH within the range of observed field values (<0.2 µM) had minimal impact on axenic cultures at ecologically relevant cell concentrations of 10^5^ cells mL^−1^, exposure to the SMC concentration of 0.8 µM HOOH was lethal to representative strains of the eMIT9312 (UH18301 and VOL1), eMED4 (VOL7) and eNATL2A (VOL3) ecotypes (Figure 2C and Figure S6). This lethality occurred for all these strains under both high (250 µmol quanta m^−2^ s^−1^, data not shown) and low (40 µmol quanta m^−2^ s^−1^, Figure 2C and Figure S6) light conditions. In contrast, when co-cultured with ecologically relevant concentrations of EZ55 (10^6^ cells mL^−1^) these strains were able to tolerate HOOH exposures at least an order of magnitude higher than the SMC value (Figure 2C and Figure S6). Indeed, the resistance of these strains in co-cultures approached that of the EZ55 helpers themselves (Figure 2C and Figure S6), well beyond any naturally occurring HOOH concentration observed in the open ocean.

### HOOH stress results in catastrophic loss of cell integrity and photosynthetic capacity

**Figure 3:**
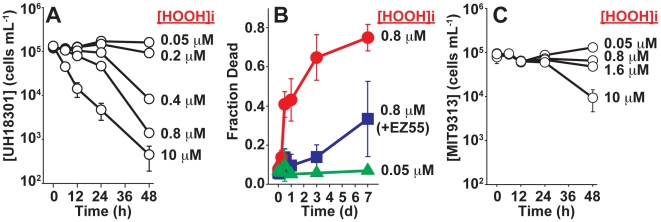
*Prochlorococcus* cell viability during HOOH exposure in unamended sterile seawater under low light. A) Change in cell concentration following exposure to the indicated [HOOH]i for axenic UH18301 in low light. B) Proportion of Sytox Green-positive (i.e. dead) UH18301 cells following exposure to the indicated [HOOH]i. Green triangles, axenic UH18301 with no added HOOH; red circles, axenic UH18301 with added HOOH; blue squares, co-culture of UH18301 with EZ55 and added HOOH. C) As in A, but for MIT9313.

The lethal effects of the SMC challenge were further characterized for axenic UH18301 in unamended seawater. Cell concentrations (detected by flow cytometry) remained constant or increased slightly during 48 h exposure to HOOH dosages up to 0.2 µM, but exposures of 0.4 µM and greater resulted in significant loss of cells (Figure 3A). At the SMC concentration of 0.8 µM, >99% of the initial UH18301 cell population was undetectable after 48 h. Moreover, as time progressed, an increasing proportion of the remaining cells stained positive for the membrane-impermeable vital stain Sytox Green (Figure 3B), strongly suggesting that cell loss is due to lysis. Sytox Green uptake was greatly attenuated in co-cultures with EZ55 (Figure 3B), suggesting that helpers protect *Prochlorococcus* against HOOH-mediated membrane permeabilization.

**Figure 4:**
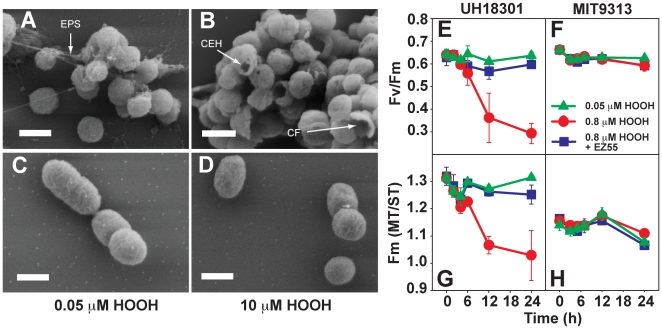
Cell envelope and photophysiological effects of HOOH on *Prochlorococcus* grown under low light. SEM of UH18301 (A and B) and MIT9313 (C and D) after 24 h exposure to 0.05 µM (A and C) or 10 µM (B and D) HOOH. All scale bars  = 500 nm. EPS, extracellular polymeric substances; CEH, cell envelope hole; CF, cell fragment. Cells were exposed to ∼10 fold higher HOOH to compensate for the ∼100 fold higher cell concentrations necessary to recover sufficient biomass for imaging. Photophysiological parameters Fv/Fm and Fm(MT/ST) (see text) of *Prochlorococcus* UH18301 (E and G) and MIT9313 (F and H) were also measured during 24 h exposure to the indicated [HOOH] in sterile unamended seawater.

Consistent with the Sytox Green results, scanning electron microscopy revealed pronounced cell envelope damage in HOOH-stressed *Prochlorococcus* cells. Untreated (≤0.05 µM HOOH) axenic UH18301 cells had the typical spherical morphology of HL *Prochlorococcus* strains, and this strain appeared to produce an extracellular matrix (Figure 4A). Damaged cells were rare, and showed signs of lysis due to osmotic stress during the fixation process. In contrast, after 24 h of exposure to HOOH, UH18301 exhibited profound envelope damage (Figure 4B). While cell size and basic shape were unchanged, cell population density was greatly diminished in these samples (e.g. Figure 3A), and no matrix of any kind was visible. Many of the intact cells had holes in their membranes, and partial fragments of cell envelopes were also observed (Figure 4B). These images strongly suggest that the decline in cell concentration upon HOOH exposure (Figure 3A) is due to a catastrophic loss of cell integrity, such that the cells lose the light-scattering and chlorophyll-based fluorescence properties used in their detection.

Concurrent with the decline in cell concentration, *Prochlorococcus* cells exposed to the SMC concentration of HOOH also suffered in their capacity for photosynthesis. Fv/Fm, a measure of photosynthetic efficiency and health, declined steadily in UH18301 cultures during 24 h of HOOH exposure, but was rescued by the presence of EZ55 helpers (Figure 4E and Figure S7). Similar results were observed for another eMIT9312 strain (VOL1), as well as representative eMED4 (VOL7) and eNATL2A (VOL3) strains, under high or low light conditions (Figure S7). Fv/Fm of control cultures (no added HOOH) was also significantly lower in cells grown under high light than under low (Figure S7). While the decrease in Fv/Fm resulting from elevated HOOH was largely eliminated by the presence of helpers, the decrease resulting from elevated light levels was not (Figure S7), which may indicate that distinct mechanisms of inhibition are at play, with the high-light associated mechanism not involving a diffusible reactive oxygen intermediary that can be removed by helpers.

The FIRe protocol used in these studies measures two distinct types of variable fluorescence. Fm(ST) is obtained using a rapid series of short flashlets to induce a single turnover of the PSII pool, whereas Fm(MT) is measured during a much longer series of flashlets, during which multiple turnovers of the system completely reduce the plastoquinone (PQ) pool. The ratio of these two values, Fm(MT/ST), thus allows an estimate of the ratio of PQ and/or other downstream components of the photosynthetic electron transport chain to photosystem II reaction centers (RCIIs) [54]. Fm(MT/ST) was significantly greater in UH18301 cultures grown in low light than in high light (Figure 4G), suggesting that under low light conditions, PQ availability increases, perhaps to maximize the efficiency of harnessing of scarce excitation energy. Following HOOH exposure, however, Fm(MT/ST) of low light acclimated cultures dropped to the level of high light acclimated cultures (Figure 4G and Figure S7). The target of this HOOH-mediated oxidative damage is not known, but whether it is the RCII itself and/or some downstream element (PQ, cytochrome b_6_f, etc.), this damage appears to result in diminished PQ re-oxidation rates and depression of Fm(MT) in relation to Fm(ST). Like Fv/Fm, however, the HOOH-mediated decline in Fm(MT/ST) is suppressed in co-cultures with EZ55 (Figure 4A). Like UH18301, all other strains assayed that are able to grow at high light (i.e., all except MIT9313) had higher Fm(MT/ST) in low light conditions. Interestingly, HOOH exposure eliminated this difference in all strains *except* VOL7 (Figure S7), suggesting that this strain may have unique, currently unknown, protective mechanisms.

### Phylogenetic distribution of helper dependency

Interestingly, while the eMIT9313 ecotype has never been observed at high abundance in the surface mixed layer [17] - most likely due to its sensitivity to high light [16] - strain MIT9313 was significantly *more* resistant to HOOH than strains of the three ecotypes (eMIT9312, eMED4, and eNATL2A) that *are* found in the HOOH-enriched surface mixed layer. MIT9313 was uniquely capable of growth at the SMC HOOH concentration (Figure S6), and this concentration had minimal impact on photosynthetic efficiency (Figure 4F, 4H, and Figure S3) or cell integrity (Figure 3C and Figure 4C–D). Importantly, none of the strains tested were able to significantly lower the HOOH concentration of the medium within the time frame of the photophysiology experiments (24 h, Figure S1), suggesting that the greater resistance of MIT9313 relative to the other strains is not achieved via higher rates of HOOH scavenging.

**Figure 5:**
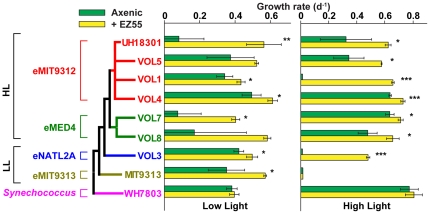
Growth at 0.2 µM HOOH as a function of helpers and light intensity for cultures with very dilute (∼100 cells mL^−1^) initial cell concentrations. Growth rates for strains representing 4 *Prochlorococcus* ecotypes and a marine *Synechococcus* ecotype with (yellow bars) or without (green bars) EZ55 helpers in low or high light (40 or 250 µmol quanta m^−2^ s^−1^, respectively). Consensus (100% support in 1000 bootstrap replicates) neighbor-joining tree was prepared using the entire rDNA operon (≈5.5 kb) and rooted on the *Synechococcus* branch. Only topology is shown; branch lengths do not represent genetic distance. Significance levels of t-tests (df = 4): *, P<0.05; **, P<0.01; ***, P<0.001. HL, high light adapted ecotypes; LL, low light adapted ecotypes.

The results with the SMC HOOH concentrations suggested that the genetically distinct ecotypes may have unique responses to oxidative stress. To explore this possibility at a finer scale, we compared their growth properties at a HOOH concentration permissive to all strains, and under a high and low light intensity that may be experienced within the surface mixed layer. Altogether, eight axenic strains of *Prochlorococcus* representing two HL and two LL ecotypes, as well as one strain of marine *Synechococcus* were tested. Invariably, axenic *Prochlorococcus* cultures inoculated at 100 cells mL^−1^ into autoclaved Pro99 (containing ∼0.2 µM HOOH) grew poorer relative to co-cultures with EZ55, with higher variability in growth rate (Figure 5) and lag time (data not shown) between replicates. In contrast, there was no difference in the growth rate or lag of *Synechococcus* WH7803 in the presence or absence of EZ55. Light intensity had variable effects on the EZ55 helping phenotype for the *Prochlorococcus* strains (Figure 5). Amongst the HL ecotypes, eMED4 strains VOL7 and VOL8 were more reliant on help in low light, whereas the members of the eMIT9312 ecotype did not share a single light preference: UH18301 was less dependent on help in high light, while VOL1 was incapable of axenic growth at this light intensity. The LL eNATL2A strain VOL3 was incapable of axenic growth under high light, whereas growth under high light was permissible in the presence of EZ55. While it exhibited relatively high HOOH resistance in low light (Figure 3C), the LL strain MIT9313 exhibited no growth after 30 days in high light from an inoculum of 100 cells mL^−1^, with or without EZ55, supporting our assertion in the prior section that photoinhibition of *Prochlorococcus* [16] includes a mechanism that is independent of diffusible HOOH.

## Discussion

### HOOH removal is the primary mechanism responsible for the helper phenotype

In this work we have confirmed our initial report [37] that heterotrophic bacteria help dilute *Prochlorococcus* cultures grow via removal of HOOH from the environment. HOOH diffuses freely across cellular membranes [55], and hence the heterotrophic HOOH degradation could occur in the cytoplasm, periplasm, or extracellular milieu, perhaps with the same overall effect. Density-dependence of HOOH resistance has also been observed in *Escherichia coli*, and catalase-positive *E. coli* has been shown to cross-protect more vulnerable catalase-negative mutants in co-culture [56]. Inter-species cross-protection from oxidative stress has been noted for heterotrophic bacterial communities involved in aromatic degradation [57] and in oral biofilms [58], as well as in a benthic mat-forming cyanobacterium [59], but to our knowledge this is the first demonstration that such helping can occur between planktonic microbes of different trophic levels.

While HOOH removal is sufficient to explain the helping phenotype of heterotrophic bacteria (at least for the EZ55 strain), other types of interactions may also contribute. For instance, heterotrophs can help eukaryotic algae by improving carbon fixation, either via increasing DIC concentrations [60] or lowering oxygen concentrations (thus diminishing the competing oxygenase reaction of Rubisco [61]). However, the addition of heterotrophic helpers to the *Prochlorococcus* cultures either led to no observable difference in bulk bicarbonate, CO_2_, or oxygen, or a change in the opposite direction to that expected for a beneficial role (Figure S2). In other cases heterotrophs help by supplying an essential metabolite (e.g. vitamin B12 [62] or indole acetic acid [63]) or trace nutrient (e.g., by producing iron-binding siderophores [64]). While we cannot rule out involvement of such cross-feeding interactions, our results suggest that any nutritional mechanism must play a secondary role relative to oxidative stress reduction, as adding HOOH back to helper treated media was alone sufficient to restore the growth limitation of dilute *Prochlorococcus* cultures (Figure 1A). Other than inorganic C, N, P, and metals, *Prochlorococcus* has no known nutritional requirements for growth [65], [66], and in fact grows better when dense versus dilute, contrary to expectations for an organism with a nutritional deficiency. Additionally, that a dense strain of *Prochlorococcus* can help a dilute strain (Figure 1B) argues against a requirement for a nutrient that *Prochlorococcus* itself cannot produce from the medium.

With the exception of MIT9313, exposure of all axenic cultures of *Prochlorococcus* to SMC levels of HOOH results in catastrophic loss of cell envelope integrity (Figure 3B and Figure 4B). Concomitant with this envelope damage is the loss of photosynthetic efficiency (Figure 4E,G), and both of these effects may be consequences of lipid peroxidation. The slow decline of HOOH in sterile, light exposed media (Figure S1) may be the result of the Fenton reaction, where photochemically produced Fe(II) reacts with HOOH to produce highly reactive OH^•^ [67]. In turn, OH^•^ can attack polyunsaturated fatty acids such as those found in most biological membranes, producing relatively stable lipid peroxide radicals that can spread via radical propagation if not countered by antioxidant defenses [55]. Both the cytoplasmic and the photosynthetic membranes may be susceptible to lipid peroxidation, and this may account for the coincident loss of cell envelope integrity and photosynthetic capacity we observed. Of note, dependence of HOOH-induced lethality on free Fe(II) could explain the greater resistance of MIT9313 to SMC challenge, as this is the only strain of *Prochlorococcus* to express the Fe-binding *dpsA* gene [4] which is linked to oxidative stress tolerance in other organisms [68]. Interestingly, the *Synechococcus* PCC7942 DpsA has a weak catalase activity [69] and if similar, the DpsA homolog of MIT9313 may be responsible for the cell's ability to eventually deplete the HOOH after 2 weeks under the SMC treatment (data not shown).

HOOH may also affect photosynthesis by impinging on the turnover of RCII protein D1. D1 proteins are continuously degraded in illuminated photosynthetic membranes, and cells must repair or replace these proteins to maintain photosynthetic activity [70]. In the freshwater cyanobacteria *Synechococcus* PCC7942 and *Synechocystis* PCC6803, exogenous HOOH inhibits protein synthesis by specific inactivation of elongation factor G [71], leading to net loss of photosystems containing functional D1. Hence, two non-mutually-exclusive mechanisms – lipid peroxidation and interruption of D1 turnover – may be responsible for the HOOH-dependent loss of photosynthetic efficiency in *Prochlorococcus*, and future studies should be aimed at testing these hypotheses.

### Impact of helpers on *Prochlorococcus* ecology

Our results suggest that *Prochlorococcus* depends on the HOOH-degrading members of the microbial community to grow in the surface mixed layer of the open ocean. Opposing photochemical production reactions [24], [25] and microbial degradation reactions [52], [53] maintain the HOOH concentration within the permissive range (<0.2 µM) for the three ecotypes of *Prochlorococcus* that exploit the mixed layer niche. Absent the mixed layer microbial community, photochemical production of HOOH yields concentrations that are lethal to all three ecotypes (Figure 2B–C). The surface monoculture (SMC) challenge (Figure 3 and Figure S6) demonstrated that, absent a counteracting microbial community, HOOH in surface seawater from the open ocean climbs to concentrations that ecologically relevant cell concentrations of these ecotypes are unable to survive. Curiously, the one strain that can tolerate the SMC challenge, MIT9313, belongs to an ecotype that is restricted from the mixed layer [17], probably due to its sensitivity to high light [16]. While the SMC challenge reflects a worst case scenario for un-helped *Prochlorococcus* (e.g. sustained presence within 5 m of the surface), we note that even modest increases of HOOH above the steady state mixed layer concentrations (e.g. 0.2 µM, Figure 2A) are enough to significantly impact growth (Figure 1, Figure 3A, and Figure 5), suggesting that the entire *Prochlorococcus* mixed layer population, not just cells at the very surface, benefit from the activity of helpers. Hence, an important but previously unrecognized role of the microbial community is to make the surface mixed layer permissive for the growth of *Prochlorococcus*, and by doing so, facilitate the expansion of its habitat range. Whether this relationship between *Prochlorococcus* and its HOOH-consuming neighbors is commensal or mutualistic remains to be determined, but as the numerically-dominant primary producer in the surface mixed layer, it is certainly conceivable that the HOOH consumers may benefit indirectly through the release of organic carbon during the growth [72] or lysis of *Prochlorococcus*.

Our study contributes to a growing body of evidence that interspecies interactions can contribute significantly to niche expansion via stress reduction. For instance, land plants in high altitude alpine sites subjected to high abiotic stress (e.g., low moisture, low temperature, strong winds) experience pronounced losses in productivity as well as increased mortality in the absence of other members of the plant community, whereas this helping effect does not occur in otherwise similar communities in less stressful, lower altitude habitats [73]. Likewise, lichenized algae and fungi gain vastly improved resistance to desiccation and oxidative stress in symbiosis, expanding the range of each to include subaerial habitats [74]. Similarly, cnidarians (e.g., corals) and zooxanthellae tolerate temperature and hypoxia extremes together that neither can tolerate separately [75]. Collectively, these studies support the broader “stress-gradient hypothesis” that cooperation should be stronger in more stressful environments [76], and emphasize the need to assess stress responses at the community level in order to understand their impacts on biological distributions in nature.

The microbial community in the surface mixed layer is genetically diverse [2], and identifying the microbes contributing most significantly to HOOH degradation is a major challenge for future studies. We know that *Prochlorococcus* degrades HOOH poorly (Figure S1) and thus is not likely to contribute significantly to HOOH decomposition in the ocean. Intriguingly, the same may be true for the most abundant heterotroph in the oligotrophic ocean as well, as the genome of *Pelagibacter ubique*, the first cultured representative of the SAR11 cluster, also lacks homologs of catalase and other antioxidant defenses and [77]. The lack of robust HOOH scavenging pathways in both the numerically dominant autotroph and heterotroph implies that this critical function may be performed by less abundant “keystone” species in the mixed layer. Given the high catalytic rate of catalase [78] and the efficiency of HOOH removal observed in culture (e.g. Figure S1), it is conceivable that a relatively small number of catalase-expressing organisms could protect the entire surface mixed layer community from solar-generated HOOH. We note that many of the confirmed helpers of *Prochlorococcus* [37] are catalase-positive members of the alpha and gamma proteobacteria whose genetic signatures appear at reasonable frequencies in the open ocean mixed layer [2], [79], and are thus potential candidates for these keystone microbes. However, there are clearly other biological mechanisms for removing HOOH (e.g., the residual HOOH scavenging abilities of catalase mutants, Table S2), any one of which may contribute to the helper phenotype of surface mixed layer communities.

### Evolutionary implications of the helper-*Prochlorococcus* interaction

Our results present an apparent paradox regarding the evolutionary history of the *Prochlorococcus* lineage: the eMIT9313 ecotype, which is restricted from the HOOH-enriched mixed layer by its sensitivity to high light, is more resistant to HOOH than the ecotypes found in high abundance in the mixed layer. eMIT9313 is the earliest branching lineage from the last common ancestor of *Prochlorococcus* and *Synechococcus* [23], [80], and although it lacks catalase, may share other ROS defense mechanisms with *Synechococcus* (e.g. *dpsA*). Indirect defense mechanisms may also be involved in eMIT9313. For example the peptidoglycan synthesis genes of MIT9313 are more similar to those of *Synechococcus* than those of the HL strain MED4, and the MIT9313 cell wall is intermediate in thickness between those of *Synechococcus* and MED4 [81]. A thicker cell wall may confer enhanced resistance by limiting HOOH diffusion into cells and/or preventing cell lysis (e.g., Figure 4B versus Figure 4D). It is not clear why eMIT9313 has retained the relatively high resistance to HOOH; perhaps the genes conferring resistance are also involved in other cellular functions that remain under selection. However, what is clear is that for the more recently derived lineages, including the LL ecotype eNATL2A, the net genomic reduction that has occurred relative to eMIT9313 [23] has coincided with a loss of HOOH resistance. Thus, the evolution of the genus leading to tolerance of temporary exposures to high light (eNATL2A) and true high light adaptation (eMED4 and eMIT9312) that allowed this lineage to exploit the surface mixed layer habitat did not coincide with a greater resistance to HOOH; in fact, the opposite occurred.

We believe that this paradox is resolved because the HOOH-consuming microbes present at the ocean's surface made the HOOH resistance genes in *Prochlorococcus* dispensable. *Prochlorococcus* evolved in the context of an extant HOOH-scavenging community, and thus as it developed tolerance to high light and possibly other environmental stresses in the surface mixed layer, it had no selection pressure to maintain the high level of resistance to HOOH. As the oligotrophic environment imposed pressure to reduce genome size [5], [22], [23], [82], [83], genes encoding these resistance mechanisms were amongst the pool of expendable genes, and were eventually lost. Similar mechanisms may have been at play during the genome streamlining of the ocean's most abundant heterotroph, *P. ubique* [84], although it is unknown as yet whether or not this organism benefits from co-culture with helpers. It is intriguing to note that both *Prochlorococcus* and *P. ubique* are conspicuously difficult to cultivate, leading to speculation that other, as-yet-uncultured organisms may be similarly dependant on helpers in nature (e.g., [64]). Hence, this study emphasizes the importance of community-level stress responses not only for the ecology, but also the evolutionary history of free-living microbes.

Finally, we note that while the catalase gene could have been lost from the *Prochlorococcus* lineage (either prior to or after the divergence from *Synechococcus*) as a consequence of neutral genetic drift, it may also have been lost as a selectively favorable event. One reason may be economic: as hypothesized for the reduction of peptidoglycan in the HL strain MED4 [81] and the reduction of phospholipids in favor of sulfolipids throughout the *Prochlorococcus* lineage [7], the loss of catalase may have been selected to lower the cell quota for scarce nutrients in the oligotrophic ocean. The catalase-peroxidase found in most cyanobacteria is a large dimeric enzyme (160 kilodaltons) that has 4 iron-containing heme co-factors [78]. If we make certain assumptions about expression levels based on published data (see Methods), we may estimate that loss of catalase-peroxidase would result in a reduction of cell quotas for Fe, P, and N by 0.2%, 0.14%, and 0.05%, respectively, for *Prochlorococcus* MED4 (Table S4). As the primary selective pressure implicated in the genomic reductions for this oligotrophic lineage is to lower the cost of such scarce nutrients for cell production [5], [22], [23], [82], [83], it is reasonable that such a gene would be lost in the absence of selective pressure for its retention. Additionally, catalases in keratinocytes have been shown to generate an unidentified form of reactive oxygen species when exposed to UV-B [85]. If similar side reactions occur for bacterial catalases, cells in the UV-exposed surface mixed layer may experience a tradeoff, degrading HOOH while also producing another form of activated oxygen; under these conditions the negative consequences may outweigh the positive ones for *Prochlorococcus*. In the future, development of robust genetic tools for *Prochlorococcus* should allow us to ectopically express catalase and determine if it indeed has a positive or negative impact under mixed layer conditions.

## Methods

### Field sites and methods

HOOH levels in the euphotic zone were measured on three cruises (Table S3): Jan-Feb 2007 in the Western Pacific (WP2), Jun-Jul 2008 in the Eastern Pacific (DCM08), and May-Jun 2009 in the Sargasso Sea (BC09). Seawater was collected in 20 L Niskin bottles and dispensed into lightproof polypropylene bottles rinsed three times with sample water. Care was taken to shield these samples from light prior to laboratory analysis. In situ HOOH levels were measured within 30 minutes of sample retrieval. HOOH measurements for WP2 were conducted using an acridinium ester chemiluminescence flow-injection method [86] implemented with a FeLume (Waterville Analytical, Waterville, ME). HOOH measurements for DCM08 and BC09 were performed using a similar protocol modified for use in an Orion-L microplate luminometer (Berthold Detection Systems, Pforzheim, Germany) (see Methods S1), and restricted to surface mixed layer (≤10 m) depths. Flow cytometry or quantitative PCR [20] confirmed that *Prochlorococcus* was abundant (≥10^4^ cells ml^−1^) in all mixed layer samples analyzed (data not shown).

To measure rates of HOOH production in sterile seawater, approximately 15 ml of the above samples were passed through 0.22 µm Millex GV syringe filters (Millipore, Billerica, MA) into custom-built UVT acrylic cuvettes, and sealed with threaded nylon caps. These cuvettes permit about 90% transmission of photosynthetically active radiation, UV-A, and UV-B, as assessed using PAR (QSL2100, Biospherical Instruments, San Diego, CA) and UV sensors (Mannix UV340, General Tools, New York, NY). Both the cuvettes and the caps were sterilized prior to use using a UV germicidal lamp. Samples in cuvettes were exposed to ambient light in a UVT acrylic deck incubator, and HOOH levels were measured at 0 and 24 h. Samples received between 60–80% of surface irradiance (i.e., ∼1200–1600 µmol quanta m^−2^ s^−1^ on clear days), and temperature was stabilized by continuous flow of surface water through the incubator. Control cuvettes were treated identically, only shielded from light.

### Cultures and culture conditions

Strains used in this study are listed in Table S1. *Prochlorococcus* cultures were grown in Pro99 natural seawater medium [66] prepared using water collected from 15 m at station R2 in the Georgia Bight (31°22.35′ N, 80°33.68′ W). Strain UH18301 was isolated from Station ALOHA (22°45′N, 158°W) in the North Pacific Subtropical Gyre, at 125 m on July 14, 2006. Seawater was diluted onto 75% Pro99 plates pre-seeded with ∼10^6^ cells mL^−1^ of helper UH06001 (*Alteromonas* sp. and a close relative of EZ55) and incubated at 22°C in a natural diel light cycle incubator with a 12∶12 light:dark cycle with a maximum irradiance of 50 µmol quanta m^−2^ s^−1^. A green *Prochlorococcus* colony that formed after ∼2 months of incubation was picked and grown in Pro99 liquid medium and this culture was later rendered clonal and axenic through the antibiotic selection method described below.

Axenic MIT9313 [87] was a generous gift of L. R. Moore, whereas the other strains (Table S1) were rendered axenic by a modified version of our previously described method [37]. Spontaneously SmR mutants of the *Prochlorococcus* strains were diluted to extinction in Falcon Microtest U-bottom 96-well microtiter plates (#35-3077, BD, Franklin Lakes, NJ) containing 200 µL Pro99 and approximately 2×10^5^ cells of the streptomycin sensitive helper *Alteromonas* sp. EZ55. These plates were incubated at 24°C in a “Sunbox” growth chamber (Percival, Perry, IA) designed to simulate a natural light regime by gradually raising light level from darkness to a “noon” maximum of 40 µmol quanta m^−2^ s^−1^ and back to darkness again over a 12/12 light/dark photoperiod [88]. Microtiter cultures exhibiting growth of *Prochlorococcus* were tested for purity (apart from the streptomycin-sensitive helper) by placing aliquots into 1/10 ProAC broth [37] containing streptomycin, followed by incubation in the dark. If this purity broth did not become turbid within a week, the Pro99 culture was diluted into fresh Pro99, grown until visibly green, and treated with 100 µg mL^−1^ streptomycin to remove the helpers. Purity of cultures was routinely confirmed by diluting aliquots of the culture into 1/10 ProAC or PLAG media [37]. Cultures were considered axenic if no turbidity formed after 60 d in purity broth, and no DAPI-stained cells other than *Prochlorococcus* were observed via fluorescence microscopy and flow cytometry.

Heterotrophic bacteria were grown in YT3 medium (per L, 10 g tryptone, 5 g yeast extract, 1 mM TAPS buffer pH 8.0, dissolved in Turk's Island Sea Salts [66] and filter sterilized with a 0.22 µm Supor filter [Pall]). Helpers were harvested by centrifugation from 2 d cultures and washed twice with sterile Pro99 prior to addition to *Prochlorococcus* cultures.

All experiments were performed at 24°C in a Sunbox (see above). “Low light” experiments were conducted in a Sunbox fitted with blue theater gels (Roscolux #69, Rosco, Stamford, CT) and set to produce a noon maximum of about 40 µmol quanta m^−2^ s^−1^; “high light” experiments used unfiltered white light with a noon maximum of about 250 µmol quanta m^−2^ s^−1^. *Prochlorococcus* strains were acclimated to growth conditions over at least two passages into fresh media prior to the start of experiments. All growth experiments used inocula taken from mid-exponential phase cultures (between 10^7^ and 10^8^ cells mL^−1^). Unless otherwise specified, all chemicals were Sigma Ultra grade (Sigma-Aldrich, St. Louis, MO).

### Quantification of Prochlorococcus and helpers

*Prochlorococcus* concentrations were determined by flow cytometry, either on a Cytopeia Influx instrument (BD, Franklin Lakes, NJ) or a Guava EasyCyte 8HT (Millipore), using established protocols [20], [89]. For some experiments, growth was tracked by bulk chlorophyll fluorescence using either a TD700 fluorometer (Turner Designs, Sunnyvale, CA, USA) equipped with an in vivo chl a filter set (excitation 340–500 nm; emission ≥665 nm) or a Synergy HT-1 96-well plate fluorometer (BioTek, Winooski, VT). In experiments using co-cultures of different *Prochlorococcus* strains, concentrations of the different populations were distinguished using quantitative PCR (qPCR) as previously described [15]. Helper cell viable counts were determined on YTSS plates containing 50 U ml^−1^ bovine liver catalase.

Exponential growth rates (µ) were calculated as the slope of the natural logarithms of at least three points during exponential phase; the highest of these measurements is C_end_, taken at time t_end_. Lag phase duration (LPD) was calculated using the equation
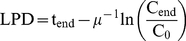
(1)where C_0_ is the initial measurement at time 0. LPD data was then expressed as an inverse lag proportion (LP^−1^), given by the ratio of LPD for a control culture (LPD_[HOOH]0_) with <0.05 µM HOOH and a culture with an elevated HOOH concentration (LPD_[HOOH]x_): 
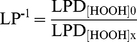
(2)such that cultures that did not grow (LPD_[HOOH]x_  =  infinity) could be assigned a plottable value of ∼0.

*Prochlorococcus* viability was determined by staining with 5 µM Sytox Green (Invitrogen, Carlsbad, CA) for at least 10 minutes prior to flow cytometry, following manufacturer's instructions. Dead cells were defined as events in the *Prochlorococcus* gate (forward angle light scatter and red fluorescence) that exhibited green fluorescence values similar to a glutaraldehyde-killed control.

### HOOH exposure experiments

The effects of elevated HOOH (i.e., different from the concentration obtained by simply autoclaving seawater) on *Prochlorococcus* were tested using cultures initially containing approximately 10^5^ cells mL^−1^ of *Prochlorococcus*, either with or without 10^6^ cells mL^−1^ of the helper heterotroph EZ55. These concentrations represent typical levels of *Prochlorococcus* and total bacteria, respectively, observed in the field [2], [3]. Seawater for the Pro99 media was prepared by ultrafiltration with a Labscale tangential flow filtration device fitted with a Pellicon XL 30 kilodalton cutoff regenerated cellulose cartridge (Millipore), followed by passage through a washed 0.22 µm Supor filter. This was necessary to produce a medium with a sufficiently low initial HOOH concentration to serve as a low HOOH control. Both autoclave and microwave sterilization led to similar HOOH levels of ∼0.2 µM (e.g., Figure S1). While filter-sterilized seawater had much lower HOOH levels (∼40±1 nM), the possible presence of *Prochlorococcus* phage in natural seawater necessitated either some form of heat sterilization prior to preparation of culture media, or else the ultrafiltration to remove virus size-class particles. *Prochlorococcus* was inoculated into Pro99 media made from this ultrafiltered seawater either unamended (<0.05 µM HOOH) or amended with HOOH (0.1 to 200 µM), and if no growth was detectable after 60 d, a culture was determined to be dead.

Experiments involving exposure to accumulating HOOH were designed using the buffer HEPES that facilitates steady, light-driven production of HOOH [42], [43]. All cultures were set to pH 8.0 and contained 3.75 mM buffer, using a combination of HEPES and TAPS [(a non-HOOH generating buffer, see Figure 1C)]) to produce a range of HOOH fluxes in a constant buffering capacity background. Cultures were acclimated to the presence of 3.75 mM buffer in TAPS-containing media before being transferred into media containing HEPES.

### Chemical and enzymatic assays

All HOOH measurements in the laboratory were performed using the Orion-L luminometer and an acridinium ester chemiluminescence protocol (Methods S1). Bulk water chemistry measurements followed standard protocols [90] using samples filtered through washed 0.22 µm Millex GV filters. Dissolved inorganic carbon (DIC) concentrations were determined by calculation based on measurements of alkalinity (by the bromcresol green endpoint method) and pH (using an UltraBasic probe, Denver Instrument, Bohemia, NY). Dissolved O_2_ (DO) was measured using an Orion 4star probe (Fisher, Waltham, MA) calibrated in water-saturated air. Dissolved organic carbon (DOC) levels were measured using a TOC-VCP analyzer (Shimadzu, Kyoto, Japan). Alkaline phosphatase activity was measured colorimetrically, following the increase in absorbance at 420 nm after addition of p-nitrophenyl phosphate (final concentration 0.04% w/v) using a BioTek Synergy HT-1 plate reader [91]. Total hydroperoxidase activity was measured by adding 0.8 µM HOOH to a sterile sample and measuring HOOH concentrations every 2 h for 6 h.

### Effects of HOOH on *Prochlorococcus* photophysiology and morphology

Photophysiological parameters were assessed using a Fast Induction and Relaxation (FIRe) fluorometer (Satlantic, Halifax, Nova Scotia) using the methodology described by Johnson [92]. Variable photosystem II fluorescence (Fv/Fm) was determined by curve fitting in MATLAB using both a rapid single turnover (ST) flash series and a longer multiple turnover (MT) series [93]. Photophysiological diel measurements were all performed beginning at the same point in the diel cycle (∼3 h after subjective “sunrise”). Initial decreases in photophysiological parameters (Figure 4E–H) were observed in all cultures, including controls, and likely represent normal diel cycling of these characteristics, as observed in *Prochlorococcus* PCC 9511 [94].

Cell morphology was assessed by scanning electron microscopy (SEM). 20 mL cultures at approximately 10^8^ cells ml^−1^ were concentrated by centrifugation at 6500 g for 15 min. Pellets were resuspended in 0.1 M cacodylate-buffered 3% glutaraldehyde for 60 min, and then washed three times with buffer water for 10 min. Cells were then post-fixed in 0.1 M cacodylate-buffered 2% osmium tetroxide for 60 min. After fixation cells were washed three times with water; during the final wash cells were allowed to settle onto a clean silicon chip. Samples were dehydrated in a graded ethanol series at 15 min intervals. Following dehydration samples were critical point dried with liquid CO_2_ in a critical point dryer (Ladd, Williston, VT). Dried samples were sputtered with a thin layer of gold before examination with a LEO 1525 scanning electron microscope (Carl Zeiss SMT Inc, Peabody, MA). As higher concentrations of cells (10^8^ cells ml^−1^) were required for the preparation of the SEM samples, we challenged the cells with 10 µM HOOH rather than the usual 0.8 µM SMC value. That an effect of this HOOH treatment was observed for the UH18301 cells but not for the more HOOH-resistant MIT9313 cells (Figure 4) indicates that the concentrations used was appropriate for these assays.

### Calculation of cell quota effects for catalase loss

Cell quotas for C, N, and P for *Prochlorococcus* MED4 were obtained from [95]. Fe quotas were obtained from [96]. By calculation the *Synechococcus* WH 7803 KatG polypeptide is 80 kD in size and contains two heme cofactors. This enzyme's active configuration is dimeric [78], so the holoenzyme is 160 kD and contains 2054 N and 4 Fe atoms. In *E. coli* there is an average of 2 mRNA copies/OTU and about 1270 copies of each expressed protein [97]. The volume of an average *E. coli* cell is 1.1 µm^3^ [98]; assuming that MED4 is spherical and 0.5 µm in diameter, its volume is 0.065 µm^3^, or about 1/17 that of *E. coli*. If we assume that the abundance ratio of an average *Prochlorococcus* protein to an average *E. coli* protein is proportional to the volume ratio of these organisms, then the average protein would be present in about 75 copies in MED4. If we further assume that catalase is present at average abundance, and that a MED4 cell contains a single chromosomal *katG* and 2 mRNA's, then loss of the *katG* gene from a putative catalase-positive MED4 ancestor would give the savings indicated in Table S4.

## Supporting Information

Methods S1 **Measurement of HOOH using a microplate luminometer.**
(DOC)

Table S1 **Strains used in this study.**
(DOC)

Table S2 **Results of helper assays in liquid Pro99.** *Prochlorococcus* was inoculated at 100 cells mL^−1^ in media with the indicated treatment.
(DOC)

Table S3 **Field sampling stations.**
(DOC)

Table S4 **Estimated effects of *katG* loss from a hypothetical catalase positive ancestor of *Prochlorococcus* MED4 on cell quotas for selected nutrients.**
(DOC)

Figure S1 **HOOH removal by** ***Prochlorococcus*** **spp. and** ***Alteromonas*** **sp. EZ55.** *Prochlorococcus* was added to autoclaved Pro99 media at the indicated initial inoculum (cells mL^−1^) either with or without 10^6^ cells mL^−1^ EZ55. Changes in [HOOH] were followed using acridinium ester chemiluminescence (Methods S1).
(TIF)

Figure S2 **Influence of** ***Prochlorococcus*** **UH18301 and** ***Alteromonas*** **EZ55 on Pro99 medium chemistry.** A) EZ55 cell concentration; B) *Prochlorococcus* UH18301 cell concentration; C) pH; D) HCO_3_^−^ concentration; E) CO_2_ concentration, representing the sum of dissolved CO_2_ and H_2_CO_3_; F) dissolved O_2_ concentration; G) Total organic carbon in 0.22 µm-filtered media. Error bars are the standard error of three biological replicates. Black circles, sterile media; green triangles, axenic UH18301; red circles, axenic EZ55; blue squares, co-cultured UH18301 and EZ55. The * in Panel B indicates that only 1 of 3 replicates survived to this point, leading to a very large standard deviation for cell counts.
(TIF)

Figure S3 **Photoinactivation of purified catalase.** Catalase was added to sterile Pro99 media at 1 U/mL and incubated in a Sunbox incubator under low light conditions (see Methods). Aliquots were removed immediately after sample preparation (circles), after 1 d (up triangles), and after 3 d (down triangles). 0.8 µM HOOH was added to these aliquots, and the change in HOOH was monitored for 6 h.
(TIF)

Figure S4 **Alkaline phosphatase activity in EZ55-treated media.** *Alteromonas* sp. EZ55 was added to Pro99 at 10^6^ cells mL^−1^ and incubated in the dark for 24 h. Alkaline phosphatase activity was measured as described in the Methods either before (solid line) or after (dashed line) 0.2 µm filtration of this medium through low-protein-binding PVDF membranes. Readings were taken every 10 min for 24 h. Error bars are the standard deviation of three replicate well on a single 96-well plate.
(TIF)

Figure S5 **Effects of chronic HOOH exposure on** ***Prochlorococcus*** **UH18301.** UH18301 was inoculated into sterile, unamended seawater containing the indicated concentration of HEPES at 10^5^ cells mL^−1^. All cultures had a constant 10 mM concentration of buffer; the difference was made up using TAPS. The control cultures for this experiment are plotted in Figure 1C. A) HOOH accumulated in proportion to the concentration of HEPES in the medium. Values are the zero-order rate constants for HOOH formation, in µM d^−1^, calculated over the first 3 d. Down triangles plot the same data shown by red circles in Figure 1C for comparison. B) Changes in cell concentration were observed over time.
(TIF)

Figure S6 **Effects of HOOH exposure on** ***Prochlorococcus*** **ecotypes.** Cultures representing 2 LL (MIT9313 and VOL3) and 2 HL (VOL1 and VOL7) ecotypes with (green circles) or without (red squares) EZ55 were exposed to the indicated [HOOH]. Cultures were inoculated at ecologically relevant concentrations (10^5^ *Prochlorococcus* and 10^6^ EZ55 cells mL^−1^) in Pro99 medium and grown under low light. Vertical red line represents the SMC HOOH concentration described in the text. LP^−1^, inverse lag proportion as described in the text and in the legend for Figure 2; *, no growth was detectable after 60 d in all 3 biological replicates.
(TIF)

Figure S7 **Photophysiological parameters of** ***Prochlorococcus*** **ecotypes following exposure to SMC (0.8 µM) HOOH exposure.** Fv/Fm and Fm(MT/ST) of representative strains of *Prochlorococcus* were measured after 24 h in sterile unamended seawater with 0.05 or 0.8 µM HOOH. Low light, maximum 40 µmol quanta m^−2^s^−1^; high light, maximum 250 µmol quanta m^−2^s^−1^. All error bars are the standard error of three biological replicates. *, axenic, HOOH-treated cultures are significantly different (t-test, df = 4, p<0.05) than both untreated axenic cultures and HOOH-treated cultures containing EZ55.
(TIF)
