# Supplementary material for: Dependence of the Cyanobacterium Prochlorococcus on Hydrogen Peroxide Scavenging Microbes for Growth at the Ocean's Surface
Source: PLoS One. 2011 Feb 3;6(2):e16805. doi: 10.1371/journal.pone.0016805 (PMC3033426; doi:10.1371/journal.pone.0016805)
Supplement: Methods S1 — Measurement of HOOH using a microplate luminometer. (DOC) [file pone.0016805.s001.doc]

Dependence of the Cyanobacterium *Prochlorococcus* on Hydrogen Peroxide

Scavenging Microbes for Growth at the Ocean’s Surface

J. Jeffrey Morris^1^, Zackary I. Johnson^2^, Martin J. Szul^1^, Martin Keller^3^, and Erik R. Zinser^1^

^1^ Department of Microbiology, University of Tennessee, Knoxville, Tennessee, United States of America

^2^ Nicholas School of the Environment, Duke University Marine Laboratory, Beaufort, North Carolina, United States of America

^3^ Oak Ridge National Laboratory, Oak Ridge, Tennessee, United States of America

SUPPORTING METHODS

*Measurement of HOOH using a microplate luminometer*

In all cases except the WP2 cruise transect, HOOH concentrations were measured using an acridinium ester chemiluminescence method [1] modified for use in an Orion-L microplate luminometer (Berthold Detection Systems, Pforzheim, Germany). 10-methyl-9-(*p*-formylphenyl)acridinium carboxylate trifluoromethanesulfonate (acridinium ester, AE) was a generous gift of M. Gonsior (University of Otago, New Zealand). Briefly, replicate 200 μL aliquots of sample were loaded onto white, flat-bottomed 96-well plates (Costar #3912, Corning Life Sciences, Lowell, Massachusetts) along with HOOH standards (2-fold dilutions ranging from 1000 nM to 15.625 nM) prepared using a seawater blank (Abyssal Blank Water, ABW) collected from 900 m depth in the oligotrophic South Pacific (32° 25’ S, 159° 5’ E), which gave a lower reading than any laboratory-prepared blank, including catalase-treated artificial seawater. The HOOH stock solution was prepared by diluting a 30% solution to approximately 25 mM, and then standardized using its absorbance at 240 nm on a DU-800 spectrophotometer (Beckman Coulter, Brea, California) using the molar absorbance coefficient 38.1 L mol^-1^ cm^-1^ [2]. Where necessary, samples were diluted in ABW to bring them into the useful range for the AE response (0-1000 nM). Additionally, samples of low salinity were diluted into ABW to compensate for the pronounced difference in clarity of seawater and deionized water after alkalinization. After loading, plates were incubated for 10 minutes in the same room as the luminometer to allow temperatures to equilibrate. The assay consisted of sequential injections of 50 μL 2 M Na_2_CO_3_ (pH 11.3) followed by 50 μL of 2.2 mg L^-1^ AE (prepared in 1 mM phosphate buffer, pH 3). Immediately after addition of AE, light output was measured for 1 s. HOOH levels were determined by comparison of experimental aliquots to a regression line prepared from the HOOH standards run on the same plate; standard deviations were calculated from the means of two technical replicates and the standard error of the slope and intercept of the standard curve.

To test the precision of this method, twelve different standard curves were run in pairs on six different days, using different batches of reagents and HOOH standards. The AE responses were linear with HOOH concentration, with an average r^2^ = 0.9988 ± 0.0006 for individual curves. The limit of detection, defined as 3 times the standard error of the blank, was 2.5 ± 2.0 nM. This value is significantly higher than the 0.35 nM limit reported by King et al. [1] for similar seawater samples using their AE method, which employed a FeLume flow injection analysis system. However, our resolution was sufficient for our purposes and the time saved by the higher throughput microplate format afforded us much more flexibility in our experimental design. On average, we could process 40 samples in 30 minutes or less, counting standard preparation and plate loading and reading. In comparison, the same number of samples and standards took us more than 2 h to process using the FeLume. Reagent usage was also much lower using the luminometer: 40 samples required less than 5 mL of AE reagent, compared to > 200 mL using the FeLume.

Initial measurements with the luminometer and the FeLume suggested that the chemiluminescence response might vary over the time required to read all the samples. We therefore ran the standard curves in pairs, one at the beginning of the reading of a plate and again at the end of the plate. While measurements for the same standards were significantly higher in the second set of readings than in the first (paired one-tailed t-test, p < 0.001), the slopes of the lines were not different (paired t-test, p > 0.5). When standard curves were made from paired measurements, they were still strongly significant (average r^2^ = 0.9986 ± 0.0005). The limit of detection increased to 7.7 ± 4.4 nM, still within an acceptable range for our experiments. Therefore, we used these paired standard curves to calculate HOOH values in all our experiments.

To confirm the specificity of the AE reaction for HOOH, at the end of one experiment all tubes were treated with 5 U mL^-1^ bovine liver catalase overnight and then assayed again. In all cases, the AE response was reduced to a level that was at or slightly below the level of the seawater blank (data not shown). We also tested the possibility that buffers in the samples (e.g., for experiments using HEPES and/or TAPS, Figure 1C) could skew estimates of HOOH by perturbing the pH of the AE reaction. Standard curves prepared either in ABW or ABW supplemented with 10 mM TAPS (pH 8.0) did not have significantly different slopes or intercepts (t-tests, P > 0.5 and 0.2, respectively).

References

1. King DW, Cooper WJ, Rusak SA, Peake BM, Kiddle JJ, et al. (2007) Flow injection analysis of H_2_O_2_ in natural waters using acridinium ester chemiluminescence: method development and optimization using a kinetic model. Anal Chem 79: 4169-4176.

2. Miller WL, Kester DR (1988) Hydrogen peroxide measurement in seawater by (*p*-hydroxyphenyl)acetic acid dimerization. Anal Chem 60: 2711-2715.
