# Supplementary material for: Dependence of the Cyanobacterium Prochlorococcus on Hydrogen Peroxide Scavenging Microbes for Growth at the Ocean's Surface
Source: PLoS One. 2011 Feb 3;6(2):e16805. doi: 10.1371/journal.pone.0016805 (PMC3033426; doi:10.1371/journal.pone.0016805)
Supplement: Table S1 — Strains used in this study. (DOC) [file pone.0016805.s002.doc]

**Table S1.** Strains used in this study.

| **Strain** | **Parent Strain** | **Relevant**  **Phenotype** | **Ecotype/Species** | **Reference^1^** | **Genome Accession Number** |
| --- | --- | --- | --- | --- | --- |
| VOL1 | MIT9215 | Sm^r 2^ | *Prochlorococcus* eMIT9312 | [1,2] | [NC_009840](http://www.ncbi.nlm.nih.gov/sites/genome?Db=genome&Cmd=ShowDetailView&TermToSearch=21422)^4^ |
| MIT9313 | MIT9313 | WT ^3^ | *Prochlorococcus* eMIT9313 | [3] | [NC_005071](http://www.ncbi.nlm.nih.gov/sites/genome?Db=genome&Cmd=ShowDetailView&TermToSearch=319) |
| VOL3 | NATL2A | Sm^r^ | *Prochlorococcus* eNATL2A | [1] and this study | [NC_007335](http://www.ncbi.nlm.nih.gov/sites/genome?Db=genome&Cmd=ShowDetailView&TermToSearch=18655)^4^ |
| VOL4 | MIT9312 | Sm^r^ | *Prochlorococcus* eMIT9312 | [1] and this study | [NC_007577](http://www.ncbi.nlm.nih.gov/sites/genome?Db=genome&Cmd=ShowDetailView&TermToSearch=18972)^4^ |
| VOL5 | AS9601 | Sm^r^ | *Prochlorococcus* eMIT9312 | [1] and this study | [NC_008816](http://www.ncbi.nlm.nih.gov/sites/genome?Db=genome&Cmd=ShowDetailView&TermToSearch=20307)^4^ |
| UH18301 | UH18301 | Sm^r^ | *Prochlorococcus* eMIT9312 | [1] and this study | MMI^5^ |
| VOL7 | MED4 | Sm^r^ | *Prochlorococcus* eMED4 | [1] and this study | [NC_005072](http://www.ncbi.nlm.nih.gov/sites/genome?Db=genome&Cmd=ShowDetailView&TermToSearch=318)^4^ |
| VOL8 | MIT9515 | Sm^r^ | *Prochlorococcus* eMED4 | [1] and this study | [NC_008817](http://www.ncbi.nlm.nih.gov/sites/genome?Db=genome&Cmd=ShowDetailView&TermToSearch=20308)^4^ |
| EZ55 | WT | WT | *Alteromonas* sp. | [2] | MMI^5^ |
| WH7803 | WT | WT | *Synechococcus* sp. | [4] | [NC_009481](http://www.ncbi.nlm.nih.gov/sites/genome?Db=genome&Cmd=ShowDetailView&TermToSearch=20997) |

^1^ Reference describing strain and/or genome

^2^ Sm^r^, spontaneous streptomycin-resistant mutant

^3^ WT, wild type

^4^  The published genome is from this strain’s streptomycin-sensitive parent.

^5^ Sequencing in progress. Data available from the Gordon and Betty Moore Foundation Marine Microbiology Initiative (<https://moore.jcvi.org/moore/>).

References

1. Kettler GC, Martiny AC, Huang K, Zucker J, Coleman ML, et al. (2007) Patterns and implications of gene gain and loss in the evolution of *Prochlorococcus*. PLoS Genet 3: 2515-2528.

2. Morris JJ, Kirkegaard R, Szul MJ, Johnson ZI, Zinser ER (2008) Facilitation of robust growth of *Prochlorococcus* colonies and dilute liquid cultures by "helper" heterotrophic bacteria. Appl Environ Microbiol 74: 4530-4534.

3. Rocap G, Larimer FW, Lamerdin J, Malfatti S, Chain P, et al. (2003) Genome divergence in two *Prochlorococcus* ecotypes reflects oceanic niche differentiation. Nature 424: 1042-1047.

4. Dufresne A, Ostrowski M, Scanlan DJ, Garczarek L, Mazard S, et al. (2008) Unraveling the genomic mosaic of a ubiquitous genus of marine cyanobacteria. Genome Biology 9: 16.
