# Supplementary material for: Dependence of the Cyanobacterium Prochlorococcus on Hydrogen Peroxide Scavenging Microbes for Growth at the Ocean's Surface
Source: PLoS One. 2011 Feb 3;6(2):e16805. doi: 10.1371/journal.pone.0016805 (PMC3033426; doi:10.1371/journal.pone.0016805)
Supplement: Table S2 — Results of helper assays in liquid Pro99. Prochlorococcus was inoculated at 100 cells mL−1 in media with the indicated treatment. (DOC) [file pone.0016805.s003.doc]

**Table S2**. Results of helper assays in liquid Pro99. *Prochlorococcus* was inoculated at 100 cells mL^-1^ in media with the indicated treatment.

|  | **Treatment** | **MIT9313** | **UH18301** |
| --- | --- | --- | --- |
| **Helper-naïve media** | **Axenic** | 186.1 ± 20.6^1^ | 189.9 ± 25.5 |
|  |  | (9/10)^2^ | (1/7) |
|  | **Catalase^3^** | -0.9 ± 6.4 | 12.1 ± 13.6 |
|  |  | (6/6) | (5/7) |
|  | **Boiled Catalase^4^** | 159.2 ± 79.3 | 153.2 ± 43.8 |
|  |  | (1/3) | (0/3) |
| **Wild-type helpers** | ***Alteromonas* sp. EZ55** | 8.3 ± 14.4 | 7.4 ± 14.9 |
|  |  | (12/12) | (9/9) |
|  | ***V. fischeri* ESR1** | 10.7 ± 19 | 73.1 ± 29.7 |
|  |  | (3/3) | (5/5) |
|  | ***S. lacuscaerulensis*** | 5.6 ± 7.8 | -1.6 ± 12.7 |
|  |  | (3/3) | (5/5) |
| **Catalase mutants** | ***Alteromonas* sp. EZ111** | 54.1 ± 34.5 | 28.9 ± 25.4 |
|  |  | (5/5) | (6/6) |
|  | ***V. fischeri* KV433** | 7.3 ± 17.4 | 79.5 ± 35.4 |
|  |  | (3/3) | (5/5) |
|  | ***S. lacuscaerulensis* EZ145** | 78.6 ± 100.8 | 207 ± 137.3 |
|  |  | (2/4) | (1/7) |

^1^ Top values, integrated daily HOOH exposure ([HOOH]) for the first week of culture, plus or minus the standard deviation of all replicates.

^2^ Bottom values in parentheses, number of cultures that survived to late exponential phase (> 10^7^ cells mL^-1^)/total number of replicates tested.

^3^ Catalase was prepared as a 1% stock of crystals (~1340 U mg^-1^ solid) in 37º C milli-Q water buffered to pH 7.0 with 10 mM phosphate buffer, then sterilized by passage through a 0.2 μm Millex GV syringe filter. Experiments were performed with a final concentration of 1 U mL^-1^ catalase.

^4^ Catalase was inactivated by heating at 95º C for 5 min.
