# Supplementary material for: Dependence of the Cyanobacterium Prochlorococcus on Hydrogen Peroxide Scavenging Microbes for Growth at the Ocean's Surface
Source: PLoS One. 2011 Feb 3;6(2):e16805. doi: 10.1371/journal.pone.0016805 (PMC3033426; doi:10.1371/journal.pone.0016805)
Supplement: Table S3 — Field sampling stations. (DOC) [file pone.0016805.s004.doc]

**Table S3.** Field sampling stations.

| **Cruise** | **Date** | **Latitude** | **Longitude** | **HOOH Method** |
| --- | --- | --- | --- | --- |
| WP2 | 1/7/07 | 12° 28.47' N | 167° 41.46' W | FeLume^1^ |
| WP2 | 1/8/07 | 10° 6.15' N | 170° 7.29' W | FeLume |
| WP2 | 1/9/07 | 7° 56.33' N | 172° 17' W | FeLume |
| WP2 | 1/10/07 | 5° 41.31' N | 174° 27.93' W | FeLume |
| WP2 | 1/14/07 | 2° 18.04' S | 177° 26.22' E | FeLume |
| WP2 | 1/15/07 | 4° 43.25' S | 174° 44.07' E | FeLume |
| WP2 | 1/17/07 | 9° 14.58' S | 169° 57.64' E | FeLume |
| WP2 | 1/20/07 | 12° 34.55' S | 169° 51.54' E | FeLume |
| WP2 | 1/23/07 | 21° 37.42' S | 169° 39.5' E | FeLume |
| WP2 | 1/26/07 | 25° 39.5' S | 165° 27.23' E | FeLume |
| WP2 | 1/27/07 | 29° 2.43' S | 164° 20.27' E | FeLume |
| WP2 | 1/28/07 | 31° 54.48' S | 163° 20.82' E | FeLume |
| WP2 | 1/29/07 | 34° 9.41' S | 162° 31.34' E | FeLume |
| WP2 | 1/30/07 | 36° 9.31' S | 161° 46.59' E | FeLume |
| WP2 | 2/2/07 | 34° 14.09' S | 160° 21.28' E | FeLume |
| DCM08 | 6/16/08 | 30° 7.28' N | 118° 24.81' W | Orion^2^ |
| DCM08 | 6/17/08 | 28° 22.81' N | 117° 16.27' W | Orion |
| DCM08 | 6/17/08 | 26° 4.22' N | 115° 46.64' W | Orion |
| DCM08 | 6/18/08 | 24° 59.35' N | 113° 24.86' W | Orion |
| DCM08 | 6/20/08 | 24° 59.93' N | 117° 59.82' W | Orion |
| DCM08 | 6/23/08 | 19° 59.97' N | 114° 0.05' W | Orion |
| DCM08 | 6/23/08 | 19° 4.15' N | 111° 20.76' W | Orion |
| DCM08 | 6/24/08 | 17° 56.64' N | 108° 5.99' W | Orion |
| DCM08 | 7/5/08 | 19° 21.85' N | 105° 22.65' W | Orion |
| DCM08 | 7/6/08 | 21° 17.99' N | 107° 48.08' W | Orion |
| BC | 5/24/09 | 30° 10.98' N | 72° 1.83' W | Orion |
| BC | 5/31/09 | 31° 39.9' N | 74° 26.4' W | Orion |
| BC | 5/31/09 | 32° 13.2' N | 75° 2.55' W | Orion |

^1^ [87]

^2^ see Methods S1
