# Supplementary material for: Dependence of the Cyanobacterium Prochlorococcus on Hydrogen Peroxide Scavenging Microbes for Growth at the Ocean's Surface
Source: PLoS One. 2011 Feb 3;6(2):e16805. doi: 10.1371/journal.pone.0016805 (PMC3033426; doi:10.1371/journal.pone.0016805)
Supplement: Table S4 — Estimated effects of katG loss from a hypothetical catalase positive ancestor of Prochlorococcus MED4 on cell quotas for selected nutrients. (DOC) [file pone.0016805.s005.doc]

**Table S4.** Estimated effects of *katG* loss from a hypothetical catalase positive ancestor of *Prochlorococcus* MED4 on cell quotas for selected nutrients.

|  | **C** | **N** | **P** | **Fe** |
| --- | --- | --- | --- | --- |
| **Total cell quota^1^** | 3.06 x 10^9^ | 3.85 x 10^8^ | 6.40 x 10^6^ | 1.51 x 10^5^ |
| **Requirements for KatG** | 350,230 | 187,620 | 8,784 | 300 |
| **% savings** | 0.01% | 0.05% | 0.14% | 0.20% |

^1^ All values are numbers of atoms per cell. Estimates were obtained based on the assumptions described in Methods.
