# Supplementary figures and images for: Dependence of the Cyanobacterium Prochlorococcus on Hydrogen Peroxide Scavenging Microbes for Growth at the Ocean's Surface
Source: PLoS One. 2011 Feb 3;6(2):e16805. doi: 10.1371/journal.pone.0016805 (PMC3033426; doi:10.1371/journal.pone.0016805)

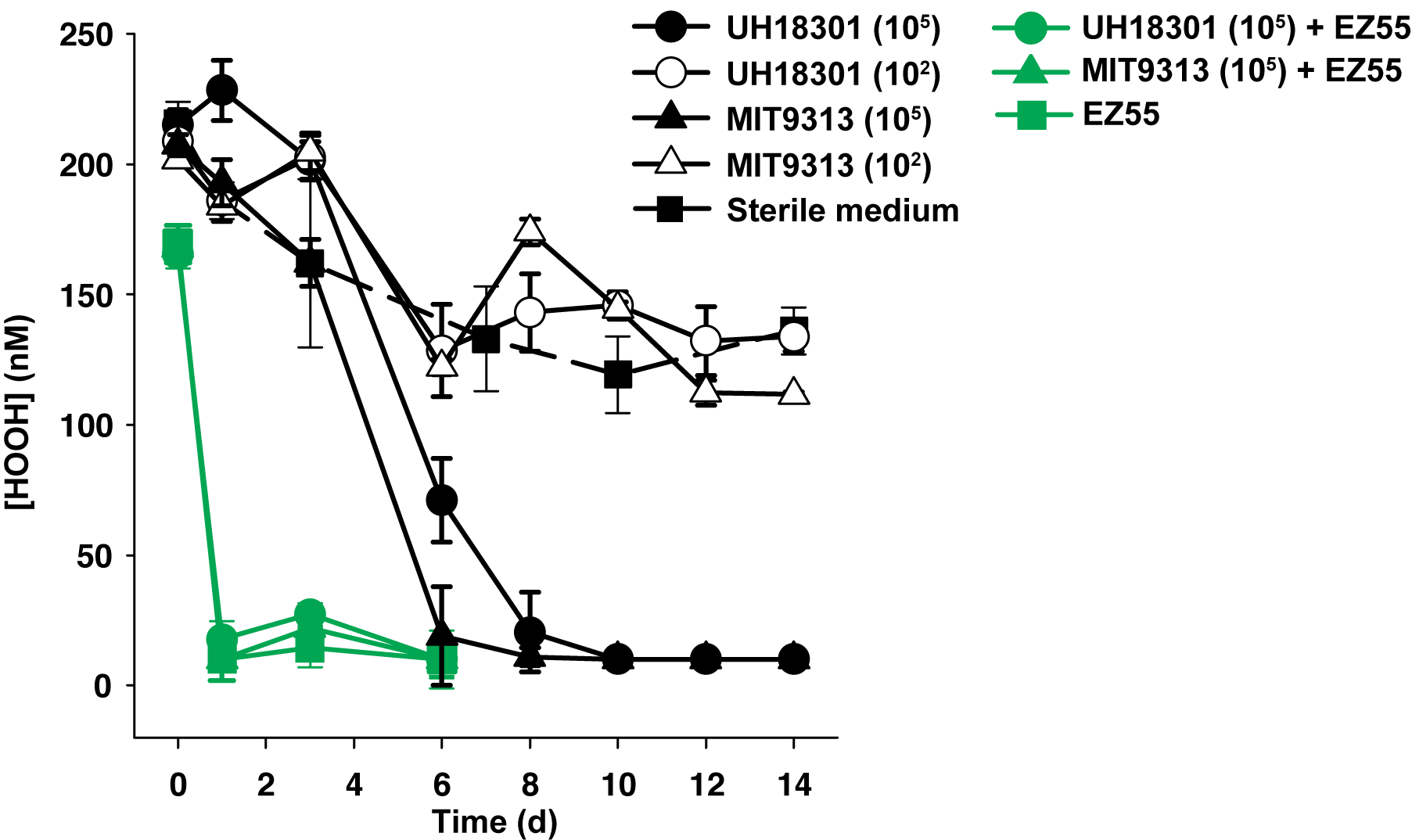

Supplement: Figure S1 — HOOH removal by Prochlorococcus spp. and Alteromonas sp. EZ55. Prochlorococcus was added to autoclaved Pro99 media at the indicated initial inoculum (cells mL−1) either with or without 106 cells mL−1 EZ55. Changes in [HOOH] were followed using acridinium ester chemiluminescence (Methods S1). (TIF) [file pone.0016805.s006.tif]

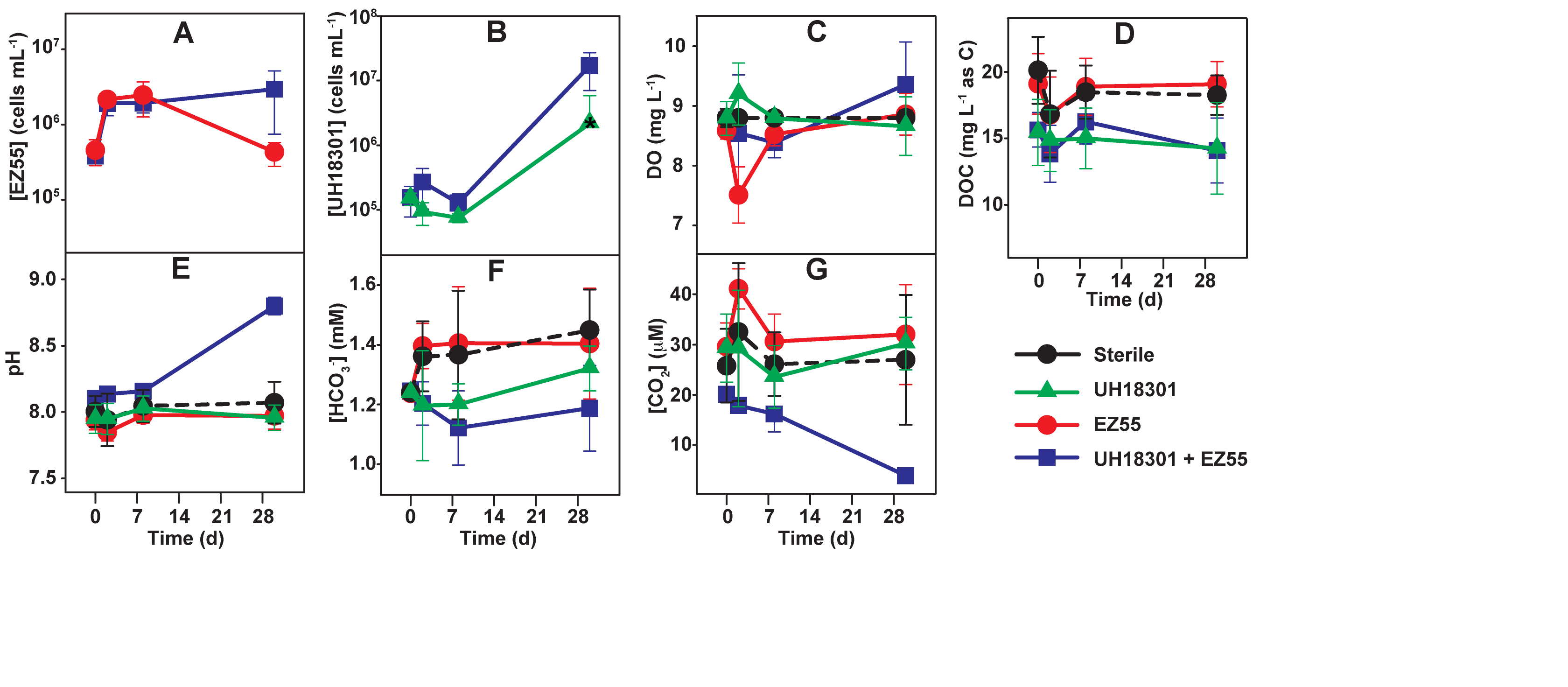

Supplement: Figure S2 — Influence of Prochlorococcus UH18301 and Alteromonas EZ55 on Pro99 medium chemistry. A) EZ55 cell concentration; B) Prochlorococcus UH18301 cell concentration; C) pH; D) HCO3 − concentration; E) CO2 concentration, representing the sum of dissolved CO2 and H2CO3; F) dissolved O2 concentration; G) Total organic carbon in 0.22 µm-filtered media. Error bars are the standard error of three biological replicates. Black circles, sterile media; green triangles, axenic UH18301; red circles, axenic EZ55; blue squares, co-cultured UH18301 and EZ55. The * in Panel B indicates that only 1 of 3 replicates survived to this point, leading to a very large standard deviation for cell counts. (TIF) [file pone.0016805.s007.tif]

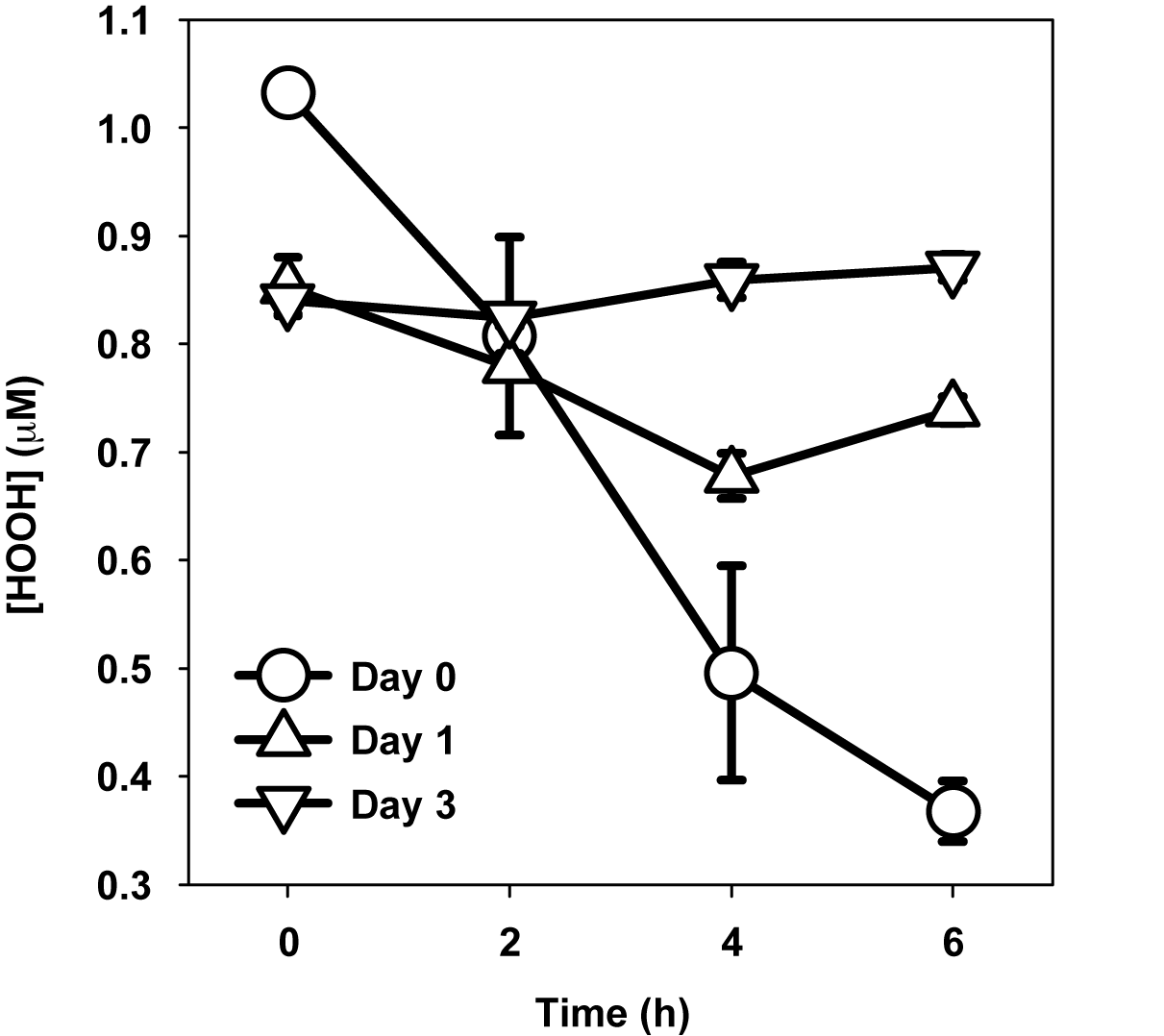

Supplement: Figure S3 — Photoinactivation of purified catalase. Catalase was added to sterile Pro99 media at 1 U/mL and incubated in a Sunbox incubator under low light conditions (see Methods). Aliquots were removed immediately after sample preparation (circles), after 1 d (up triangles), and after 3 d (down triangles). 0.8 µM HOOH was added to these aliquots, and the change in HOOH was monitored for 6 h. (TIF) [file pone.0016805.s008.tif]

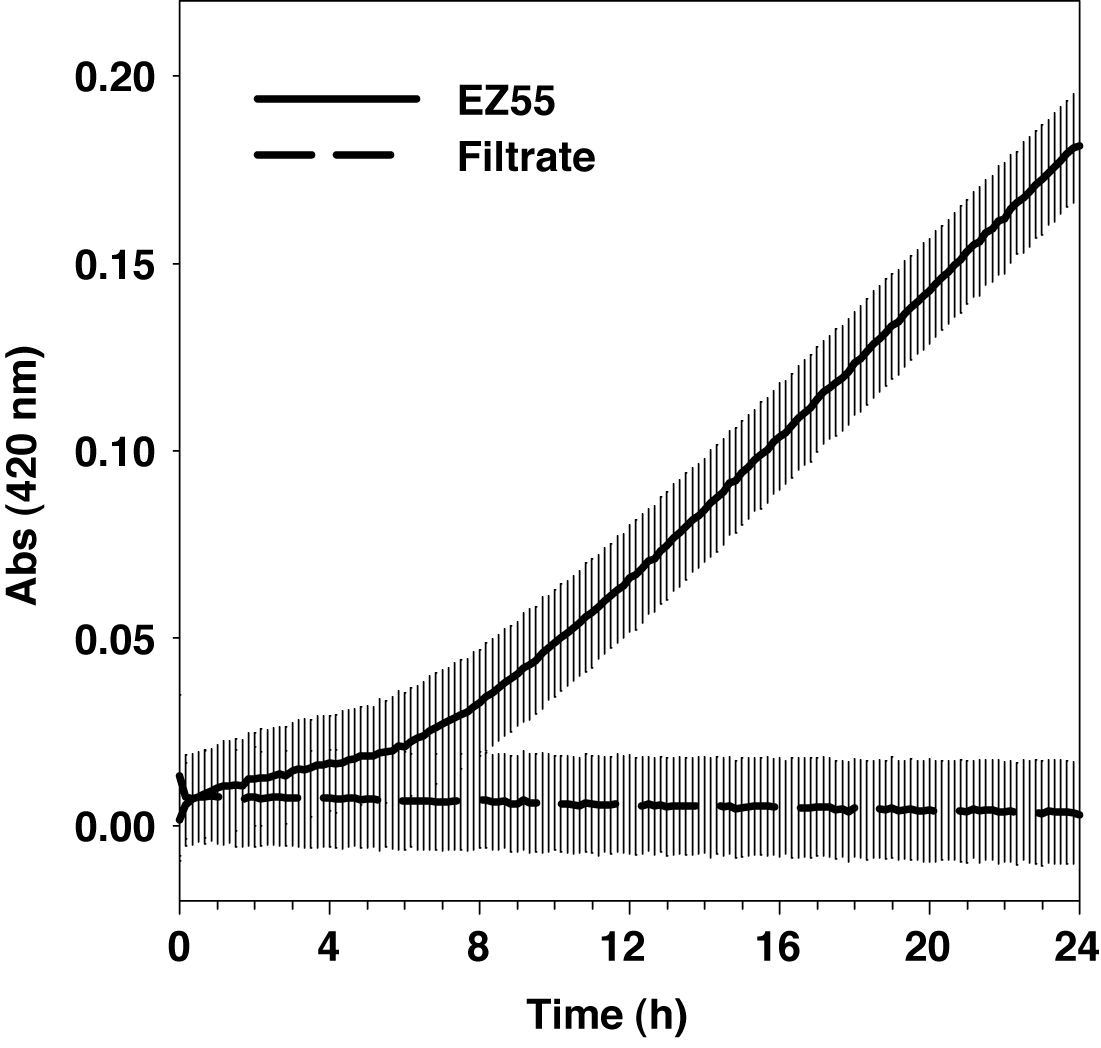

Supplement: Figure S4 — Alkaline phosphatase activity in EZ55-treated media. Alteromonas sp. EZ55 was added to Pro99 at 106 cells mL−1 and incubated in the dark for 24 h. Alkaline phosphatase activity was measured as described in the Methods either before (solid line) or after (dashed line) 0.2 µm filtration of this medium through low-protein-binding PVDF membranes. Readings were taken every 10 min for 24 h. Error bars are the standard deviation of three replicate well on a single 96-well plate. (TIF) [file pone.0016805.s009.tif]

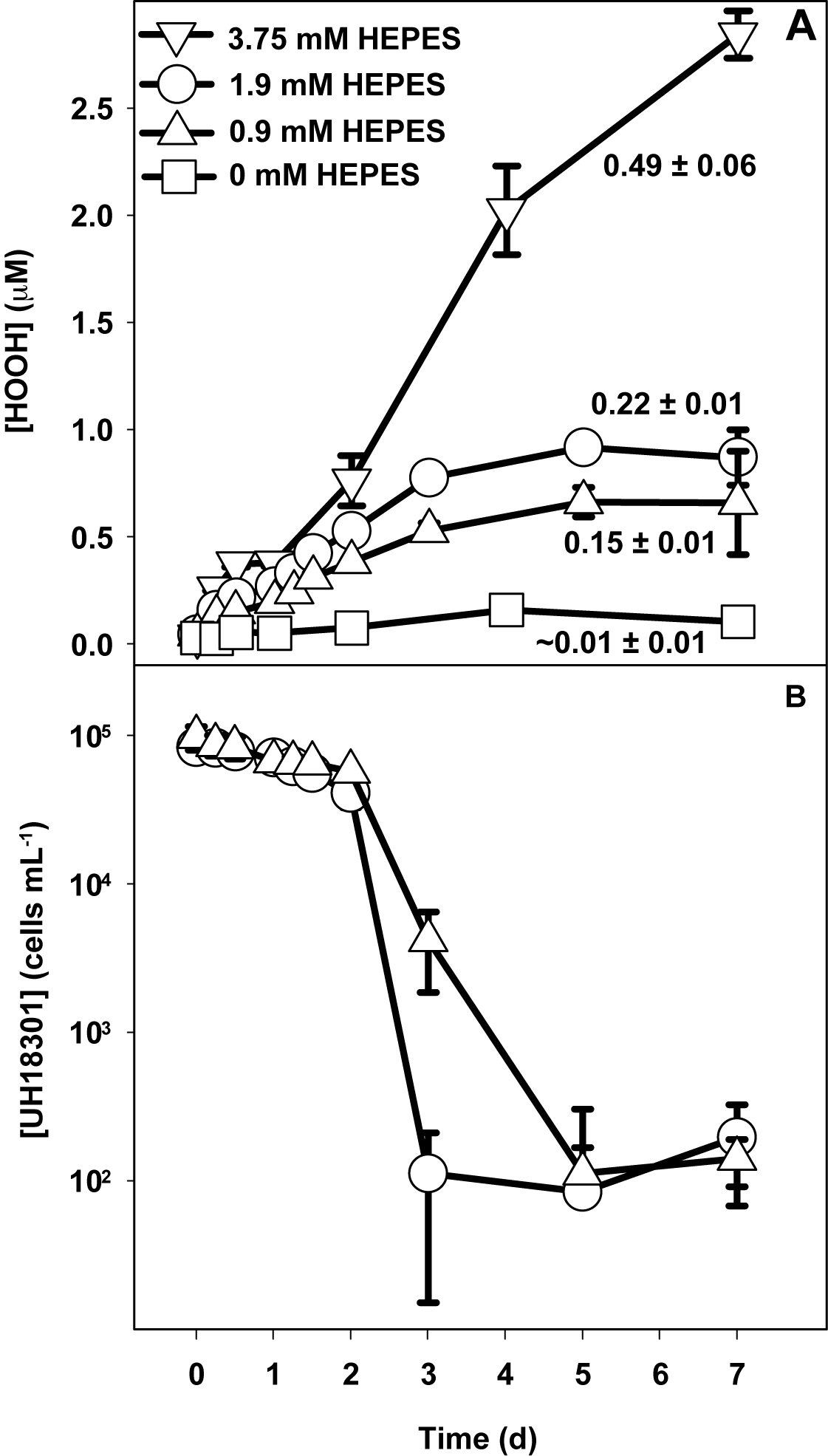

Supplement: Figure S5 — Effects of chronic HOOH exposure on Prochlorococcus UH18301. UH18301 was inoculated into sterile, unamended seawater containing the indicated concentration of HEPES at 105 cells mL−1. All cultures had a constant 10 mM concentration of buffer; the difference was made up using TAPS. The control cultures for this experiment are plotted in Figure 1C. A) HOOH accumulated in proportion to the concentration of HEPES in the medium. Values are the zero-order rate constants for HOOH formation, in µM d−1, calculated over the first 3 d. Down triangles plot the same data shown by red circles in Figure 1C for comparison. B) Changes in cell concentration were observed over time. (TIF) [file pone.0016805.s010.tif]

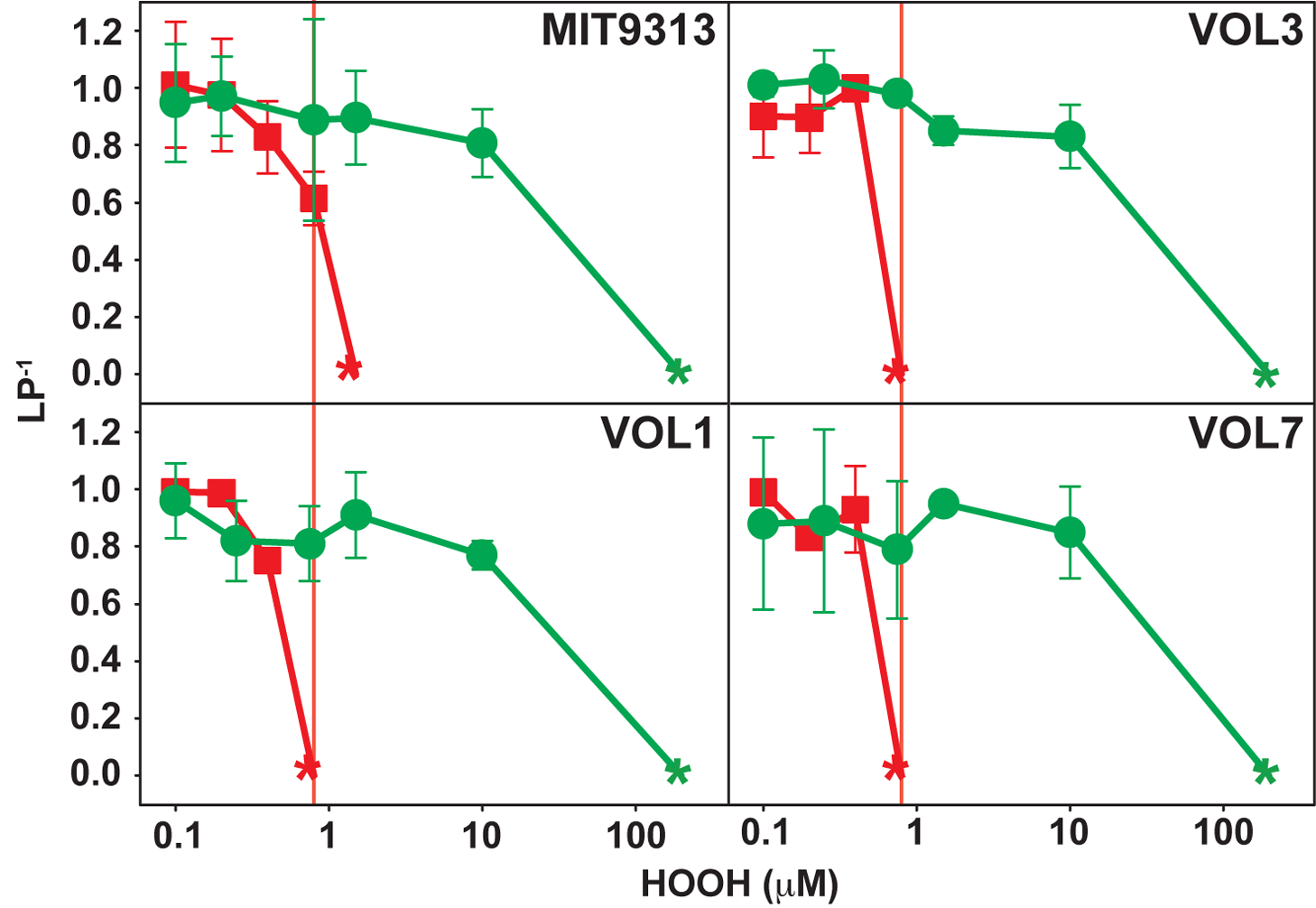

Supplement: Figure S6 — Effects of HOOH exposure on Prochlorococcus ecotypes. Cultures representing 2 LL (MIT9313 and VOL3) and 2 HL (VOL1 and VOL7) ecotypes with (green circles) or without (red squares) EZ55 were exposed to the indicated [HOOH]. Cultures were inoculated at ecologically relevant concentrations (105 Prochlorococcus and 106 EZ55 cells mL−1) in Pro99 medium and grown under low light. Vertical red line represents the SMC HOOH concentration described in the text. LP−1, inverse lag proportion as described in the text and in the legend for Figure 2; *, no growth was detectable after 60 d in all 3 biological replicates. (TIF) [file pone.0016805.s011.tif]

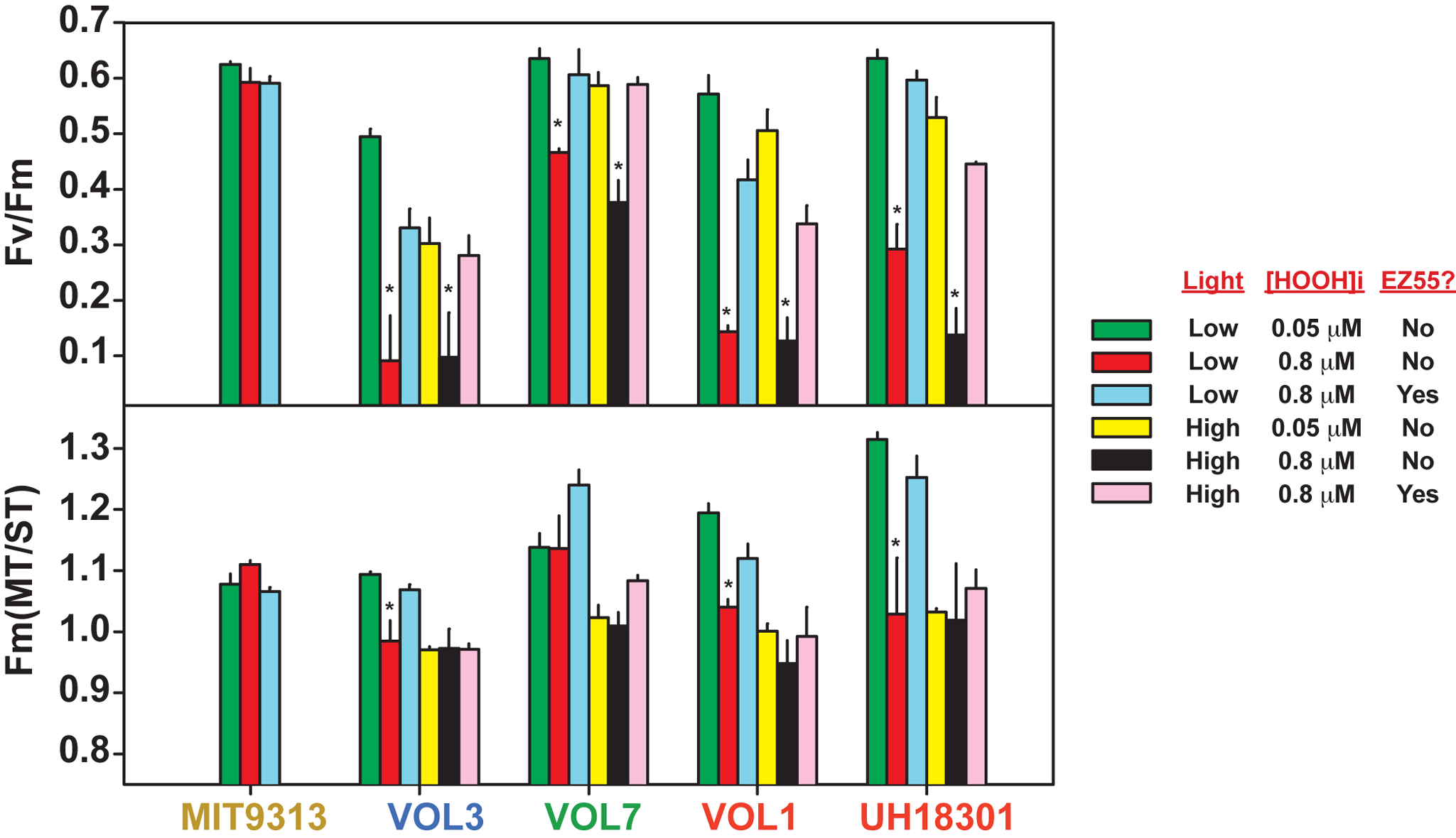

Supplement: Figure S7 — Photophysiological parameters of Prochlorococcus ecotypes following exposure to SMC (0.8 µM) HOOH exposure. Fv/Fm and Fm(MT/ST) of representative strains of Prochlorococcus were measured after 24 h in sterile unamended seawater with 0.05 or 0.8 µM HOOH. Low light, maximum 40 µmol quanta m−2s−1; high light, maximum 250 µmol quanta m−2s−1. All error bars are the standard error of three biological replicates. *, axenic, HOOH-treated cultures are significantly different (t-test, df = 4, p<0.05) than both untreated axenic cultures and HOOH-treated cultures containing EZ55. (TIF) [file pone.0016805.s012.tif]
